# Supplementary material for: High-Throughput Screen Reveals the Structure–Activity Relationship of the Antimicrobial Lasso Peptide Ubonodin
Source: ACS Cent Sci. 2023 Mar 1;9(3):540–50. doi: 10.1021/acscentsci.2c01487 (PMC10037499; doi:10.1021/acscentsci.2c01487)
Supplement: Supplementary file 1 — oc2c01487_si_001.pdf [file oc2c01487_si_001.pdf]

A High-Throughput Screen Reveals the Structure-Activity Relationship of the Antimicrobial Lasso Peptide Ubonodin

Supporting Information

Alina Thokkadam[1], Truc Do[1], Xinchun Ran[2], Mark P. Brynildsen[1,3], Zhongyue J. Yang[2,4-6], A. James Link[1,3,7,*]

[1]Department of Chemical and Biological Engineering, Princeton University, Princeton, NJ 08544, United States

[2]Department of Chemistry, Vanderbilt University, Nashville, TN 37235, United States

[3]Department of Molecular Biology, Princeton University, Princeton, NJ 08544, United States

[4]Department of Chemical and Biomolecular Engineering, Vanderbilt University, Nashville, TN 37235, United States

[5]Data Science Institute, Vanderbilt University, Nashville, TN 37235, United States

[6]Vanderbilt Institute of Chemical Biology, Vanderbilt University, Nashville, TN 37235, United States

[7]Department of Chemistry, Princeton University, Princeton, NJ 08544, United States

*Corresponding author: ajlink@princeton.edu

Table of Contents

Methods…………………………………………………………..…...……………...….... Pages S3-S9

Supporting Figures………………..…………...………………………………………...Pages S10-S30

Supporting Tables………………………………………………………………………..Pages S31-S44

Supporting References…………………………………………………………………...Page S45

Methods

Safety

No unexpected or unusually high safety hazards were encountered.

Cloning

All cloning was conducted by PCR amplification (using Q5 polymerase), restriction digestion, ligation, (using T4 DNA ligase), and transformation into chemically competent *E. coli* XL1-Blue. All cloning with a pUboABC backbone was done by plating transformation mixtures onto Lysogeny Broth (LB) plates supplemented with 100 µg/mL ampicillin and 0.2% w/v glucose, and colonies were inoculated into LB media supplemented with 100 µg/mL ampicillin and 0.2% w/v glucose to suppress any leaky expression. Cloning for all other plasmid constructs was done by plating transformation mixtures onto Lysogeny Broth (LB) plates supplemented with 100 µg/mL ampicillin, and colonies were inoculated into LB media supplemented with 100 µg/mL ampicillin. Plasmids were extracted using Qiagen QIAprep Spin Miniprep Kit and sequence verified using Sanger sequencing. All primer sequences are listed in Table S7, and all plasmid constructs are listed in Table S8.

pUboABC was cloned by amplifying pWC99 with pQE-80 *Nhe*I For and pAT8 *Nco*I Rev. The amplicon was ligated into pWC99 digested with *Nhe*I and *Nco*I.

pUboBC was cloned by digesting pUboABC with *Nhe*I and *Nco*I and ligating that fragment into pQE-80 digested with *Nhe*I and *Nco*I.

pUboABC S5H I16P and pUboABCD S5H I16P were cloned with overlap PCR using pWC99 as the template. One fragment was amplified with pQE-80 *Eco*RI For and S5H I16P Rev v2 and the second fragment was amplified with S5H I16P For v2 and pQE-80 *Hin*dIII Rev. The fragments were joined with a second round of PCR and was amplified with pQE-80 *Eco*RI For and pQE-80 *Hin*dIII Rev. pUboABC S5H I16P was cloned by ligating the amplicon into pUboBC digested with *Eco*RI and *Hin*dIII. pUboABCD S5H I16P was cloned by ligating the insert into pWC99 digested with *Eco*RI and *Hin*dIII.

All remaining plasmid constructs were cloned with overlap PCR. One fragment was amplified with *Xho*I For Lib and a mutagenic reverse primer and the second fragment was amplified with *Nhe*I Rev Lib and a mutagenic forward primer. The templates and mutagenic primers for each plasmid construct are listed in Table S8. The fragments were joined with a second round of PCR and amplified with *Xho*I For Lib and *Nhe*I Rev Lib. Amplicons were ligated into the appropriate plasmid backbone digested with *Xho*I and *Nhe*I-HF. The plasmid backbones used for each plasmid construct are listed in Table S8.

Spot Dilution Assay

The appropriate plasmid was transformed into electrocompetent *E. coli* MC1061, plated onto LB plates supplemented with 100 µg/mL ampicillin and 0.2% w/v glucose, and incubated at 30 °C for 16 hours. Colonies were inoculated into LB media supplemented with 100 µg/mL ampicillin and 0.2% w/v glucose and grown at 37 °C for 16 hours. These cultures were sub-cultured to an OD_{600} of 0.02 in 50 mL of LB media supplemented with 100 µg/mL ampicillin in a 250 mL flask. Once the OD_{600} reached 0.3, a 1 mL aliquot was sampled immediately prior to inducing the cultures with 100 µM isopropyl β-D-1-thiogalactopyranoside (IPTG). The aliquot was washed with Phosphate-Buffered Saline (PBS) two times and ten-fold serial dilutions were spotted onto LB plates supplemented with 100 µg/mL ampicillin and 0.2% w/v glucose. For each following timepoint, a 1 mL sample of each culture was withdrawn, washed with PBS two times, and ten-

fold serial dilutions were spotted onto LB plates supplemented with 100 μg/mL ampicillin and 0.2% w/v glucose. Plates were incubated for 16 hours at 37 °C before imaging.

Colony-Forming Units of *E. coli* MC1061 pUboABC and *E. coli* MC1061 pUboBC

pUboABC and pUboBC were transformed into electrocompetent *E. coli* MC1061, plated onto LB plates supplemented with 100 μg/mL ampicillin and 0.2% w/v glucose, and incubated at 37 °C for 16 hours. Single colonies were inoculated into LB media supplemented with 100 μg/mL ampicillin and 0.2% w/v glucose and shaken at 37 °C for 16 hours. These cultures were then sub-cultured to an OD_{600} of 0.02 into 50 mL of LB media supplemented with 100 μg/mL ampicillin in a 250 mL flask. Ten-fold serial dilutions of the culture were plated onto LB plates supplemented with 100 μg/mL ampicillin and 0.2% w/v glucose. The cultures were then grown at 37 °C, and once the OD_{600} reached 0.3, a 1 mL sample of the culture was withdrawn immediately before the cultures were induced with 10 μM or 100 μM IPTG and returned to grow longer. The 1 mL sample was washed with PBS two times and ten-fold serial dilutions were plated onto LB plates supplemented with 100 μg/mL ampicillin and 0.2% w/v glucose. For each following timepoint, a 1 mL sample of each culture was withdrawn, washed with PBS two times, and ten-fold serial dilutions were plated onto LB plates supplemented with 100 μg/mL ampicillin and 0.2% w/v glucose. All plates were incubated at 37 °C for 16 hours, after which the number of colonies was counted. Three biological replicates of this assay were done.

Co-Culture of *E. coli* MC1061 pUboABC and *E. coli* MC1061 pUboBC

pUboABC (0.9 ng) and pUboBC (0.1 ng) were transformed into electrocompetent *E. coli* MC1061 with a one-hour outgrowth at 37 °C in LB media supplemented with 0.2% w/v glucose. A small quantity of DNA was used to reduce the probability of two plasmids being transformed into the same cell. The transformation mixture was plated onto an LB plate supplemented with 100 μg/mL ampicillin and 0.2% w/v glucose and incubated at 37 °C for 16 hours, yielding at least 1,000 colonies. Colonies were resuspended in LB media supplemented with 100 μg/mL ampicillin and sub-cultured to an OD_{600} of 0.02 into 50 mL of LB media supplemented with 100 μg/mL ampicillin in a 250 mL flask. Plasmids were extracted from the remaining resuspended colony mixture using a Qiagen QIAprep Spin Miniprep Kit and labeled as the MC1061 transformation sample.

The culture was grown until OD_{600} reached 0.3 at which point 1 mL samples were aliquoted and washed with PBS two times prior to plating dilutions onto LB plates supplemented with 100 μg/mL ampicillin and 0.2% w/v glucose. The culture was then split into two flasks of 25 mL each; one flask was induced with 10 μM IPTG and another flask was induced with 100 μM IPTG. Subsequent timepoints were taken by aliquoting 1 mL samples from the cultures, washing with PBS two times, and plating dilutions onto LB plates supplemented with 100 μg/mL ampicillin and 0.2% w/v glucose. Plates were incubated at 30 °C for 16 hours; each sample had at least 10,000 colonies. Colonies were resuspended in LB media supplemented with 100 μg/mL ampicillin and 0.2% w/v glucose, and plasmids were extracted using a Qiagen QIAprep Spin Miniprep Kit. Each plasmid sample, as well as a sample of 90% pUboABC and 10% pUboBC, was PCR amplified. Each PCR reaction contained 100 ng of template DNA and was amplified with *Xho*I For and *Nhe*I Rev primers for 10 cycles. Reactions were then run on a 1.5% agarose gel. Two biological replicates of this assay were done.

Library Construction: Single Mutants

Single-site saturation mutagenesis (SSM) libraries were created with overlap PCRs using Q5 polymerase for each of the 26 mutated positions in the ubonodin core peptide (all positions excluding the Gly1 and Glu8 residues). The PCR template was pWC99 treated with Exonuclease

V to reduce genomic DNA contamination. One fragment was amplified with *Xho*I For Lib and a mutagenic reverse primer and the second fragment was amplified with *Nhe*I Rev Lib and a mutagenic forward primer. The mutagenic primers for each residue-specific library are listed in Table S9. A low annealing temperature of 55 °C was used to promote binding of mutagenic primers. The fragments were joined with a second round of PCR and amplified with *Xho*I For Lib and *Nhe*I Rev Lib. A residue-specific library hereafter refers to the portion of the library that was cloned with one set of mutagenic primers. For example, the G2 residue-specific library refers to the portion of the library that was cloned using the G2 Lib For and G2 Lib Rev primers.

Amplicons were digested with *Nhe*I-HF and *Xho*I using restriction digestion and ligated into pUboABC that had been digested with *Nhe*I-HF and *Xho*I and treated with Antarctic Phosphatase. Digested inserts were stored at -20 °C for next-generation sequencing (NGS). Ligation mixtures were directly transformed into chemically competent *E. coli* XL1-Blue, plated onto LB plates supplemented with 100 µg/mL ampicillin and 0.2% w/v glucose, and incubated at 37 °C for 16 hours, leading to at least 320 colonies per residue mutated (yielding 10-fold coverage of all nucleotide mutants). Colonies were subsequently resuspended in 5 mL of LB media supplemented with 100 µg/mL ampicillin and plasmids were extracted using a Qiagen QIAprep Spin Miniprep Kit. All residue-specific libraries were cloned separately, then pooled at equimolar levels and sequenced with an Illumina MiSeq Micro 300nt. Data on the pooled samples indicated that a few G2 variants were missing (pUboABC G2F, G2I, G2M, G2N, G2W, and G2Y), so plasmids encoding these variants were individually cloned, then added to the library such that all residue-specific libraries were still present at roughly equimolar levels. This sample is hereafter referred to as the single mutant library.

Library Construction: Double Mutants

Single-site saturation mutagenesis (SSM) libraries were created with overlap PCRs using Q5 polymerase for each of the ubonodin core peptide positions to be mutated (at residues Asp3, Ser5, Asn11, Arg12, Pro13, Met14, His15, Ile16, His17, Asp18, Trp19, Gln20, Ile21, Met22, and Asp23). The PCR template was the single mutant library treated with Exonuclease V to reduce genomic DNA contamination. One fragment was amplified with *Xho*I For Lib and a mutagenic reverse primer and the second fragment was amplified with *Nhe*I Rev Lib and a mutagenic forward primer. The mutagenic primers for each residue-specific library are listed in Table S9. A low annealing temperature of 55 °C was used to promote binding of mutagenic primers. The fragments were joined with a second round of PCR and amplified with *Xho*I For Lib and *Nhe*I Rev Lib. Amplicons were digested with *Nhe*I-HF and *Xho*I using restriction digestion and ligated into pUboABC that had been digested with *Nhe*I-HF and *Xho*I and treated with Antarctic Phosphatase. Digested inserts were stored at -20 °C for NGS. Ligation mixtures were then desalted using a 0.025 µM nitrocellulose membrane in water. Desalted ligation mixtures were transformed into freshly prepared electrocompetent *E. coli* DH5α with a one-hour outgrowth in Super Optimal broth with Catabolite repression (SOC) at 37 °C. Transformation mixtures were plated onto LB plates supplemented with 100 µg/mL ampicillin and 0.2% w/v glucose, and incubated at 30 °C for 16 hours, yielding at least 120,000 colonies per residue mutated (yielding 10-fold coverage of all nucleotide mutants). Colonies were subsequently resuspended in 15 mL of LB media supplemented with 100 µg/mL ampicillin, and plasmids were extracted using a Qiagen QIAprep Spin Miniprep Kit. Residue-specific libraries were then combined in equimolar amounts, constituting the double mutant library.

Screen Methodology: MiSeq Sequencing

One ng of the single mutant library was transformed into freshly prepared electrocompetent *E. coli* MC1061 cells with a one-hour outgrowth in LB media supplemented with 0.2% w/v glucose at 37 °C. A small quantity of DNA was used to reduce the probability of two

plasmids being transformed into the same cell. Transformation mixtures were plated onto LB plates supplemented with 100 µg/mL ampicillin and 0.2% w/v glucose and incubated at 30 °C for 16 hours to obtain ~3.7 x 10^5 colonies (at least 8 x 10^3 colonies were needed to obtain at least 10-fold coverage of all nucleotide mutants). Colonies were resuspended in LB media supplemented with 100 µg/mL ampicillin. Each library was sub-cultured to an OD_{600} of 0.02 into a 250 mL flask containing 50 mL of LB media supplemented with 100 µg/mL ampicillin. Plasmids were extracted from the remaining resuspended colony mixture using a Qiagen QIAprep Spin Miniprep Kit and labeled as the screen transformation sample.

The 50 mL cultures were grown at 37 °C until the OD_{600} reached 0.3. A 1 mL aliquot was sampled from the culture, washed with PBS two times, and plated onto LB plates supplemented with 100 µg/mL ampicillin and 0.2% w/v glucose. Immediately after aliquoting, the culture was induced with 100 µM IPTG and returned to the shaker. One hour after induction, a 1 mL sample was aliquoted from the culture, washed with PBS two times, and dilutions were plated onto LB plates supplemented with 100 µg/mL ampicillin and 0.2% w/v glucose. The plates were incubated at 30 °C for 16 hours, yielding at least 4.9 x 10^4 colonies for all timepoints (at least 8 x 10^3 colonies were needed to obtain at least 10-fold coverage of all nucleotide mutants). The colonies from each plate were resuspended in 15 mL of LB media supplemented with 100 µg/mL ampicillin, and plasmids were extracted using a Qiagen QIAprep Spin Miniprep Kit.

Screen Methodology: NovaSeq Sequencing

One ng each of the single mutant library and double mutant library were transformed into freshly prepared electrocompetent *E. coli* MC1061 cells with a one-hour outgrowth in LB media supplemented with 0.2% w/v glucose at 37 °C. A small quantity of DNA was used to reduce the probability of two plasmids being transformed into the same cell. Transformation mixtures were plated onto LB plates supplemented with 100 µg/mL ampicillin and 0.2% w/v glucose and incubated at 30 °C for 16 hours to obtain ~1.4 x 10^6 colonies for the single mutant library and ~2.1 x 10^6 colonies for the double mutant library (at least 8 x 10^3 colonies were needed for the single mutant library and at least 2 x 10^6 colonies were needed for the double mutant library to obtain at least 10-fold coverage of all nucleotide mutants). Colonies were resuspended in LB media supplemented with 100 µg/mL ampicillin. Each library was sub-cultured to an OD_{600} of 0.02 into a 250 mL flask containing 50 mL of LB media supplemented with 100 µg/mL ampicillin. Plasmids were extracted from the remaining resuspended colony mixture using a Qiagen QIAprep Spin Miniprep Kit and labeled as the screen transformation sample.

The 50 mL cultures were grown at 37 °C until the OD_{600} reached 0.3. One mL aliquots were sampled from each culture, washed with PBS two times, and plated onto LB plates supplemented with 100 µg/mL ampicillin and 0.2% w/v glucose. Each culture was then split into two 250 mL flasks with 25 mL of culture in each; one flask was induced with 10 µM IPTG and another flask was induced with 100 µM IPTG. The following timepoints were taken: 1 hour after induction for both induction conditions, and 2 hours and 3 hours after induction for the 10 µM IPTG condition. The timepoints were taken by aliquoting 1 mL samples from the cultures, washing with PBS two times, and plating dilutions onto LB plates supplemented with 100 µg/mL ampicillin and 0.2% w/v glucose. The plates were incubated at 30 °C for 16 hours, yielding at least 4.2 x 10^4 colonies for all timepoints for the single mutant library and at least 7.8 x 10^6 colonies for all time points for the double mutant library (at least 8 x 10^3 colonies were needed for the single mutant library and at least 2 x 10^6 colonies were needed for the double mutant library to obtain at least 10-fold coverage of all nucleotide mutants). The colonies from each plate were resuspended in 15 mL of LB media supplemented with 100 µg/mL ampicillin, and plasmids were extracted using a Qiagen QIAprep Spin Miniprep Kit.

Next-Generation Sequencing: Library Preparation

Each sample was PCR amplified with Q5 polymerase and 10 amplification cycles. Primers amplified between the Gly2 and Gly28 DNA region of the plasmids. Both forward and reverse primers had an adaptor sequence. Reverse primers (all primers in Table S7 that begin with "P7") also had a barcode specific to each sample. The forward primer, P5-AT1F, was used for all samples but the reverse primers were different for each sample. The primer sequences are listed in Table S7. The DNA was purified using a Zymo DNA Clean & Concentrator. The single mutant library was initially sequenced using an Illumina MiSeq Micro 300nt. When sequencing on the Illumina NovaSeq 6000 Sequencing System, the samples from the screen were combined such that each single mutant library sample represented 1% of the total DNA and each double mutant library sample represented 11.5% of the total DNA to achieve sufficient coverage of the larger double mutant library.

Next-Generation Sequencing: Processing Raw Data

The sequencing results were demultiplexed using Barcode Splitter 0.18.4.0 in Galaxy. The demultiplexed DNA sequencing reads in FASTA format were further processed on the Princeton Della server using the custom Python code NGS-code-annotated.py that can be found at the Link lab Github page: https://github.com/ajlinklab/Ubolib . This code iterated through each unique DNA sequence that was read and directionally compared it to the wild-type ubonodin DNA sequence (5'-GGCGATGGCAGCATTGCGGAATACTTTAACCGTCCGATGCATATTCATGATTGGCAGATTATGGATAGCGGCTATTATGGC-3') and the wild-type ubonodin amino acid sequence (N-GDGSIAEYFNRPMHIHDWQIMDSGYYG-C) to identify any mutations and their locations. Due to trimming of sequencing reads, note that the DNA sequence starts at the second codon and the amino acid sequence starts at the second amino acid. For comparison of amino acid sequences, the original DNA sequence that was read was first translated using the standard codon table within the Biopython package. Note that if multiple mutations were found in a single amino acid sequence, the mutations and their counts were recorded as a single entity (e.g., the S5T E8D double mutant appears 140 times in total across the reads found in the current FASTA file). By contrast, comparison of the DNA sequence was conducted on a codon-by-codon basis and multiple codon mutations that were found in a single DNA sequence were recorded as separate entities (e.g., the GGC10GTC ATT16TTT double mutant is recorded as GGC7GTC and ATT13TTT each appearing 89 times in total across the reads found in the current FASTA file). Identified mutations and their counts were saved as tab-delimited output text files. The data is deposited in NCBI as BioProject number PRJNA894114.

Next-Generation Sequencing: Additional Data Analysis

For the single mutant library samples sequenced with the NovaSeq, all amino acid variants with >500 reads were kept, and for the double mutant library samples sequenced with the NovaSeq, all amino acid variants with 10 reads or higher were kept. For the single mutant library sequenced samples with the MiSeq, all amino acid variants with 40 reads or higher were kept, except for the post-IPTG sample in which all amino acid variants with 10 reads or higher were kept. The frequency of each variant in each sample was calculated using Equation S1.

Equation S1: $frequency = \frac{Number\ of\ reads\ of\ variant}{Total\ number\ of\ reads\ in\ sample} \times 100\%$

Relative frequencies were calculated to compare the change in frequency of a variant throughout the screen using Equation S2. Line graphs were constructed using relative frequency values and plotted using Excel.

Equation S2: $Relative\ frequency = \frac{Frequency\ of\ variant\ in\ sample}{Frequency\ of\ variant\ at\ cloning\ transformation}$

The enrichment was calculated to compare the change in frequency of a variant from the cloning transformation to another sample using Equation S3. Variants that increased in frequency had positive enrichment values and variants that decreased in frequency had negative enrichment values.

Equation S3: $enrichment = \log_2 \frac{frequency\ of\ variant\ [specific\ sample]}{frequency\ of\ variant\ [cloning\ transformation]}$

Histograms were constructed using MATLAB. The dropout ratio was calculated using Equation S4. Excess kurtosis was calculated in MATLAB using Equation S5 where *μ* is the mean of *x*, *σ* is the standard deviation of *x*, and *E*(*t*) represents the expected value of the quantity *t*.

Equation S4: $dropout\ ratio = \log_2 \frac{Number\ of\ variants\ at\ mode\ (excluding\ dropout\ variants)}{Number\ of\ dropout\ variants}$

Equation S5: $excess\ kurtosis = \frac{E(x-\mu)^4}{\sigma^4} - 3$

Frequency of residue-specific variants were determined for the single mutant library samples using Equation S6:

Equation S6: $frequency\ of\ residue\ variants = \frac{\sum Reads\ for\ all\ variants\ of\ a\ residue}{Total\ number\ of\ reads\ in\ sample} \times 100\%$

Non-clustered heatmaps were constructed using MATLAB. In order for the dropout variants to affect the clustering in the clustered heatmaps, the dropout variants in the clustered single mutant heatmaps were arbitrarily assigned an enrichment value of -10 and the dropout variants in the clustered double mutant heatmap were arbitrarily assigned an enrichment value of -20. The pheatmap package in R was used to construct the clustered single mutant heatmaps and the NG-CHM[1] package in R was used to construct the clustered double mutant heatmap. Euclidean distances were used for hierarchical clustering of heatmaps.

The appropriate files for DeepLasso are contained in DeepLasso.zip (which contains the Python code for DeepLasso, the training dataset, and the test dataset). Notably, the preprocessing code "preprocess.py" and the trainer.train module of "train.py" were modified from DLKcat.[2]

Expression and Purification of Ubonodin Variants

pUboABCD variants were transformed into electrocompetent *E. coli* BL21 cells and grown in M9 minimal media supplemented with 40 μg/mL of each canonical amino acid, 0.5 μg/mL thiamine, and 100 μg/mL ampicillin at 37 °C. Once the OD_{600} reached 0.2, cultures were induced with 1 mM IPTG and grown at 20 °C for 20 hours. Cultures were centrifuged at 4,000 g for 20 minutes, and the supernatant was applied to a Thermo Fischer HyperSep 6 mL C8 column. The column was activated with 6 mL of methanol and washed with 12 mL of water prior to applying the supernatant to the column. The column was then washed with 12 mL of water and the extract was eluted with 6 mL of methanol. The methanol was evaporated with a rotary evaporator prior to being resuspended in 1 mL of 75/25 water/acetonitrile per 1 L of culture. Supernatant extracts were injected onto an HPLC using water and acetonitrile with 0.1% trifluoroacetic acid in which 0-1 min ran 10% acetonitrile, 1-20 min ran 10-50% acetonitrile with a linear gradient, and 20-25 min ran 50-90% acetonitrile with a linear gradient. Fractions were collected using the collection windows specified in Table S10 and lyophilized. Injecting these HPLC fractions onto an LC-MS

indicated that ubonodin N11W and ubonodin N11W H17T were expressed at levels too low to continue purification.

Ubonodin A7P, ubonodin R12F, ubonodin H17G, ubonodin A7G N11M, ubonodin A7P I16A, and ubonodin R12V H17G were determined to be pure after purifying with the previously stated HPLC gradient. Ubonodin M14N, ubonodin I16D, ubonodin I16E, ubonodin R12F W19G, ubonodin S5T I16D, and ubonodin I16E D23A required a second round of HPLC purification. The lyophilized fractions were resuspended in 75/25 water/acetonitrile and injected onto an HPLC using water and acetonitrile with 0.1% trifluoroacetic acid in which 0-1 min ran 10% acetonitrile, 1-20 min ran 10-28% acetonitrile with a linear gradient, and 20-25 min ran 28-48% acetonitrile with a linear gradient. Ubonodin S5H I16P also required a second round of HPLC purification. The lyophilized fraction was resuspended in 75/25 water/acetonitrile and injected onto an HPLC using water and acetonitrile with 0.1% trifluoroacetic acid in which 0-2 min ran 10% acetonitrile, 2-20 min ran 10-30% acetonitrile with a linear gradient, and 20-25 min ran 30-90% acetonitrile with a linear gradient. Collection windows are specified in Table S10. Purity was verified by injecting pure samples onto an LC-MS, and peptides were resuspended in the solvent appropriate for their hydrophobicity, as specified in Table S10.

Antimicrobial Assay

The *Burkholderia* strains used were *B. cenocepacia* AU0756, and *B. cenocepacia* AU24326. The strains were grown at 32 °C. Streaked plates of *B. cenocepacia* AU0756 were grown for 48 hours while streaked plates of *B. cenocepacia* AU24326 were grown for 72 hours.

Broth microdilution assays were conducted following guidelines provided by the Clinical & Laboratory Standards Institute (CLSI). Two to three colonies from a streaked plate were inoculated into 5 mL of LB media and grown for 16 hours. The dense cultures were then sub-cultured at a 1:100 dilution in 5 mL of LB media and grown until the cultures reached the mid-exponential stage (OD_{600} 0.4-0.6). The cultures were then sub-cultured to OD_{600} 0.0005 in cation-adjusted Mueller Hinton broth along with two-fold serial dilutions of ubonodin variants for a final volume of 100 µL in a 96-well plate. Since acetonitrile inhibits *Burkholderia* growth in liquid media, ubonodin variants that were originally suspended in 50/50 water/acetonitrile were lyophilized and resuspended in PBS. This resulted in the concentrated peptide stocks being relatively clear suspensions and were thoroughly mixed before adding to the 96-well plates. The plates were grown at 32 °C for 16 hours while shaking at 250 rpm before measuring the OD_{600}.

A

```
BurkholderiaRNAPbeta 1     MQYSFTEKKRIRKSFAKRPIVHQVPFLLATQLESFSTFLQADVPATQRKPEGLQAAFTSVFPIVSHNGFARLEFVSYALS 80
                           M YS+TEKKRIRK F KRP V  VP+LL+ QL+SF  F++ D P  Q    GL+AAF SVFPI S++G + L++VSY L
EcoliRNAPbeta        1     MVYSYTEKKRIRKDFGKRPQVLDVPYLLSIQLDSFQKFIEQD-PEGQ---YGLEAAFRSVFPIQSYSGNSELQYVSYRLG 76

BurkholderiaRNAPbeta 81    APAFNIKECQQRGLTYCSALRAKVRLVILDKESPNKPVVKEVKEQEVYMGEIPLMTPTGSFVINGTERVIVSQLHRSPGV 160
                            P F+++ECQ RG+TY + LR K+RLVI ++E+P +  VK++KEQEVYMGEIPLMT  G+FVINGTERVIVSQLHRSPGV
EcoliRNAPbeta        77    EPVFDVQECQIRGVTYSAPLRVKLRLVIYEREAP-EGTVKDIKEQEVYMGEIPLMTDNGTFVINGTERVIVSQLHRSPGV 155

BurkholderiaRNAPbeta 161   FFEHDKGKTHSSGKLLFSARIIPYRGSWLDFEFDPKDILYFRVDRRRKMPVTILLKAIGLTPEQILANFFVFDNFTLMDE 240
                           FF+ DKGKTHSSGK+L++ARIIPYRGSWLDFEFDPKD L+ R+DRRRK+P TI+L+A+  T EQIL  FF    F + D
EcoliRNAPbeta        156   FFDSDKGKTHSSGKVLYNARIIPYRGSWLDFEFDPKDNLFVRIDRRRKLPATIILRALNYTTEQILDLFFEKVIFEIRDN 235

BurkholderiaRNAPbeta 241   GAQLEFVPERLRGEVARFDITDRDGKVIVQKDKRINAKHIRDLEAAKTKFISVPEDYLLGRVLAKNVVDGDTGEVIASAN 320
                             Q+E VPERLRGE A FDI + +GKV V+K +RI A+HIR LE    K I VP +Y+ G+V+AK+ +D  TGE+I +AN
EcoliRNAPbeta        236   KLQMELVPERLRGETASFDI-EANGKVYVEKGRRITARHIRQLEKDDVKLIEVPVEYIAGKVVAKDYIDESTGELICAAN 314

BurkholderiaRNAPbeta 321   DEVTESVLEKLREAGIKDIQTLYTNDLDQGPYISSTLRVDETTDKTAARIAIYRMMRPGEPPTEEAVEALFNRLFYSEEA 400
                            E++  +L KL ++G K I+TL+TNDLD GPYIS TLRVD T D+ +A + IYRMMRPGEPPT EA E+LF  LF+SE+
EcoliRNAPbeta        315   MELSLDLLAKLSQSGHKRIETLFTNDLDHGPYISETLRVDPTNDRLSALVEIYRMMRPGEPPTREAAESLFENLFFSEDR 394

BurkholderiaRNAPbeta 401   YDLSKVGRMKFNRRVSRDEITGPMTLQDDDILATIKILVELRNGKGEVDDIDHLGNRRVRCVGELAENQFRAGLVRVERA 480
                           YDLS VGRMKFNR + R+EI G   L  DDI+  +K L+++RNGKGEVDDIDHLGNRR+R VGE+AENQFR GLVRVERA
EcoliRNAPbeta        395   YDLSAVGRMKFNRSLLREEIEGSGILSKDDIIDVMKKLIDIRNGKGEVDDIDHLGNRRIRSVGEMAENQFRVGLVRVERA 474

BurkholderiaRNAPbeta 481   VKERLGQAESENLMPHDLINSKPISSAIREFFGSSQLSQFMDQTNPLSEITHKRRVSALGPGGLTRERAGFEVRDVHPTH 560
                           VKERL    + + LMP D+IN+KPIS+A++EFFGSSQLSQFMDQ NPLSEITHKRR+SALGPGGLTRERAGFEVRDVHPTH
EcoliRNAPbeta        475   VKERLSLGDLDTLMPQDMINAKPISAAVKEFFGSSQLSQFMDQNNPLSEITHKRRISALGPGGLTRERAGFEVRDVHPTH 554

BurkholderiaRNAPbeta 561   YGRVCPIETPEGPNIGLINSLALYAHLNEYGFLETPYRKVVDSKVTDQIDYLSAIEEGRYMIAQANAAIDENGTLIDELV 640
                           YGRVCPIETPEGPNIGLINSL++YA  NEYGFLETPYRKV D  VTD+I YLSAIEEG Y+IAQAN+ +DE G  +++LV
EcoliRNAPbeta        555   YGRVCPIETPEGPNIGLINSLSVYAQTNEYGFLETPYRKVTDGVVTDEIHYLSAIEEGNYVIAQANSNLDEEGHFVEDLV 634

BurkholderiaRNAPbeta 641   SSREAGETMMVTPDRIQYMDVAPSQIVSVAASLIPFLEHDDANRALMGSNMQRQAVPCLRPEKPVVGTGIERTCAVDSGT 720
                           + R  GE+ + + D++ YMDV+  Q+VSV ASLIPFLEHDDANRALMG+NMQRQAVP LR +KP+VGTG+ER  AVDSG
EcoliRNAPbeta        635   TCRSKGESSLFSRDQVDYMDVSTQQVVSVGASLIPFLEHDDANRALMGANMQRQAVPTLRADKPLVGTGMERAVAVDSGV 714

BurkholderiaRNAPbeta 721   TVQAFRGGVVDYVDAGRIVIRVNDDEAVAGEVGVDIYNLIKYTRSNQNTNINQRPIVKMGDKVSRGDVLADGASTDLGEL 800
                           T  A RGGVV YVDA RIVI+VN+DE   GE G+DIYNL KYTRSNQNT INQ P V +G+ V RGDVLADG STDLGEL
EcoliRNAPbeta        715   TAVAKRGGVVQYVDASRIVIKVNEDEMYPGEAGIDIYNLTKYTRSNQNTCINQMPCVSLGEPVERGDVLADGPSTDLGEL 794

BurkholderiaRNAPbeta 801   ALGQNMLIAFMPWNGYNFEDSILISEKVVADDRYTSIHIEELNVVARDTKLGPEEITRDISNLAEVQLGRLDESGIVYIG 880
                           ALGQNM +AFMPWNGYNFEDSIL+SE+VV +DR+T+IHI+EL  V+RDTKLGPEEIT DI N+ E  L +LDESGIVYIG
EcoliRNAPbeta        795   ALGQNMRVAFMPWNGYNFEDSILVSERVVQEDRFTTIHIQELACVSRDTKLGPEEITADIPNVGEAALSKLDESGIVYIG 874

BurkholderiaRNAPbeta 881   AEVEAGDVLVGKVTPKGETQLTPEEKLLRAIFGEKASDVKDTSLRVPSGMSGTVIDVQVFTREGIQRDKRAQQIIDDELK 960
                           AEV  GD+LVGKVTPKGETQLTPEEKLLRAIFGEKASDVKD+SLRVP+G+SGTVIDVQVFTR+G+++DKRA +I + +LK
EcoliRNAPbeta        875   AEVTGGDILVGKVTPKGETQLTPEEKLLRAIFGEKASDVKDSSLRVPNGVSGTVIDVQVFTRDGVEKDKRALEIEEMQLK 954

BurkholderiaRNAPbeta 961   RYRLDLNDQLRIVEGDAFQRLARMLVGKVANGGPKKLAKGTKIDQAYLEDLDHYHWFDIRLADDEAAASLEAIKNSIEEK 1040
                           + + DL+++L+I+E   F R+  +LV     GG         ++    L+ L    W ++ L D+E     LE +    +E
EcoliRNAPbeta        955   QAKKDLSEELQILEAGLFSRIRAVLVA----GG---------VEAEKLDKLPRDRWLELGLTDEEKQNQLEQLAEQYDEL 1021

BurkholderiaRNAPbeta 1041  RHQFDLAFEEKRKKLTQGDELPPGVLKMVKVYLAVKRRLQPGDKMAGRHGNKGVVSKIVPIEDMPYMADGRPADVVLNPL 1120
                           +H+F+    E KR+K+TQGD+L PGVLK+VKVYLAVKRR+QPGDKMAGRHGNKGV+SKI PIEDMPY  +G P D+VLNPL
EcoliRNAPbeta        1022  KHEFEKKLEAKRRKITQGDDLAPGVLKIVKVYLAVKRRIQPGDKMAGRHGNKGVISKINPIEDMPYDENGTPVDIVLNPL 1101

BurkholderiaRNAPbeta 1121  GVPSRMNVGQVLEVHLGWAAKGLGWRIGEMLQRQAKIEELRVFLTKIYNESG--RQE-DLESFTDDEILELAKNLREGVP 1197
                           GVPSRMN+GQ+LE HLG AAKG+G +I  ML++Q ++ +LR F+ + Y+     RQ+ DL +F+D+E++ LA+NLR+G+P
EcoliRNAPbeta        1102  GVPSRMNIGQILETHLGMAAKGIGDKINAMLKQQQEVAKLREFIQRAYDLGADVRQKVDLSTFSDEEVMRLAENLRKGMP 1181

BurkholderiaRNAPbeta 1198  FATPVFDGATEEEMGKMLDLAFPDDIAEQLGMNPSKNQVRLYDGRTGEMFERRVTLGYMHYLKLHHLVDDKMHARSTGPY 1277
                            ATPVFDGA E E+ ++L           +LG  P+  Q+RLYDGRTGE FER VT+GYM+ LKL+HLVDDKMHARSTG Y
EcoliRNAPbeta        1182  IATPVFDGAKEAEIKELL----------KLGDLPTSGQIRLYDGRTGEQFERPVTVGYMYMLKLNHLVDDKMHARSTGSY 1251

BurkholderiaRNAPbeta 1278  SLVTQQPLGGKAQFGGQRFGEMEVWALEAYGASYVLQEMLTVKSDDVTGRTKVYENLVKGDHVIDAGMPESFNVLVKEIR 1357
                           SLVTQQPLGGKAQFGGQRFGEMEVWALEAYGA+Y LQEMLTVKSDDV GRTK+Y+N+V G+H ++ GMPESFNVL+KEIR
EcoliRNAPbeta        1252  SLVTQQPLGGKAQFGGQRFGEMEVWALEAYGAAYTLQEMLTVKSDDVNGRTKMYKNIVDGNHQMEPGMPESFNVLLKEIR 1331

BurkholderiaRNAPbeta 1358  SLGIDIDLD 1366
                           SLGI+I+L+
EcoliRNAPbeta        1332  SLGINIELE 1340
```

Similarity = 80.28%
Identity = 65.96%

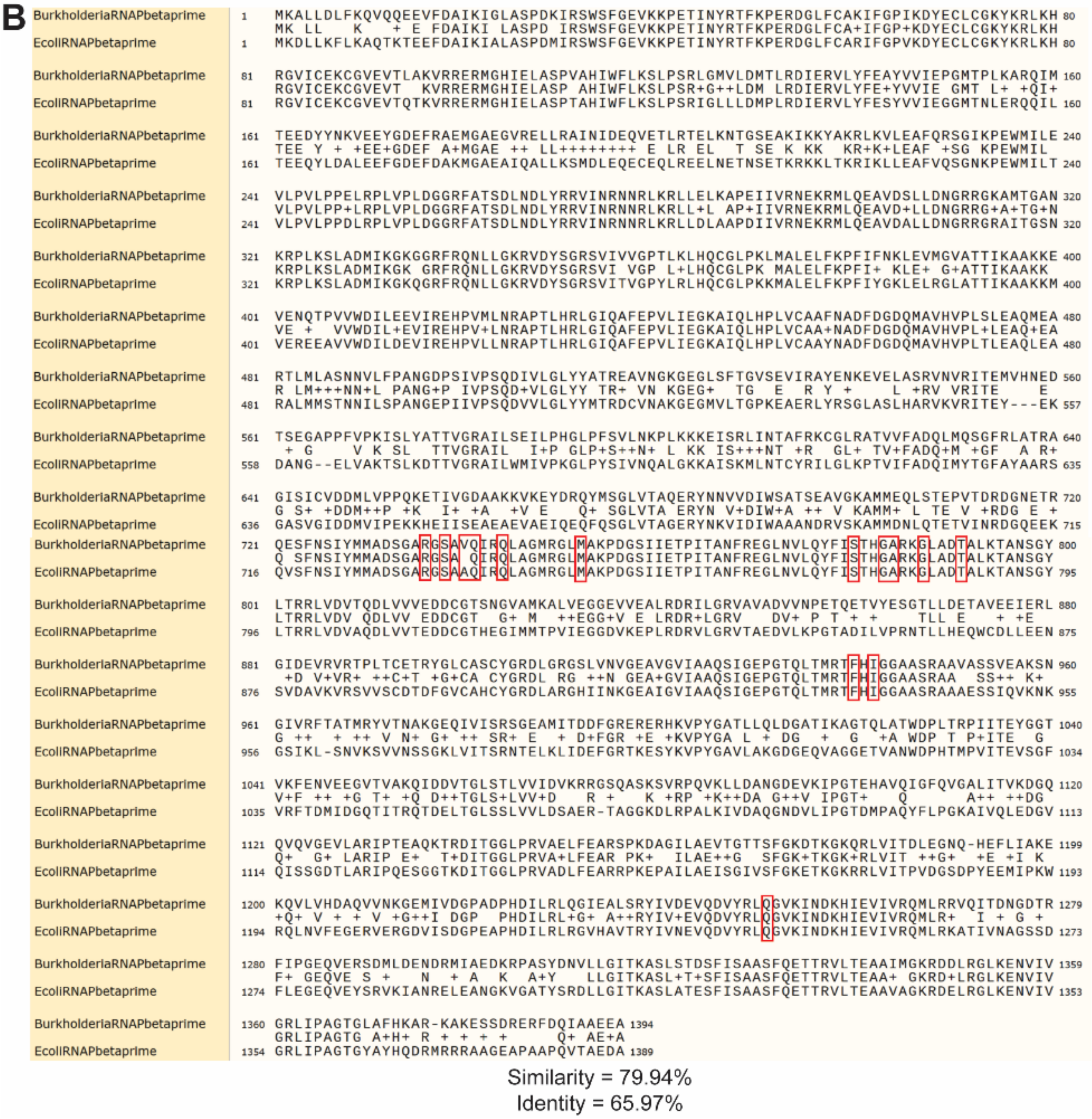

Figure S1. Alignment of the *Burkholderia cepacia* RNAP subunits with the *E. coli* RNAP subunits that have been shown to interact with microcin J25,[3] **A**: the β subunit and **B**: the β’ subunit. Residues that directly interact with microcin J25 (D675, N677, R678, S1105, R1106, and M1107 in the β subunit and R731, S733, A735, Q736, Q739, M747, S775, G778, A779, G782, T786, F935, I937, and Q1225 in the β’ subunit) are boxed in red. All these residues are conserved in the *B. cepacia* RNAP subunits except for A735 in the β’ subunit which is substituted to valine instead.

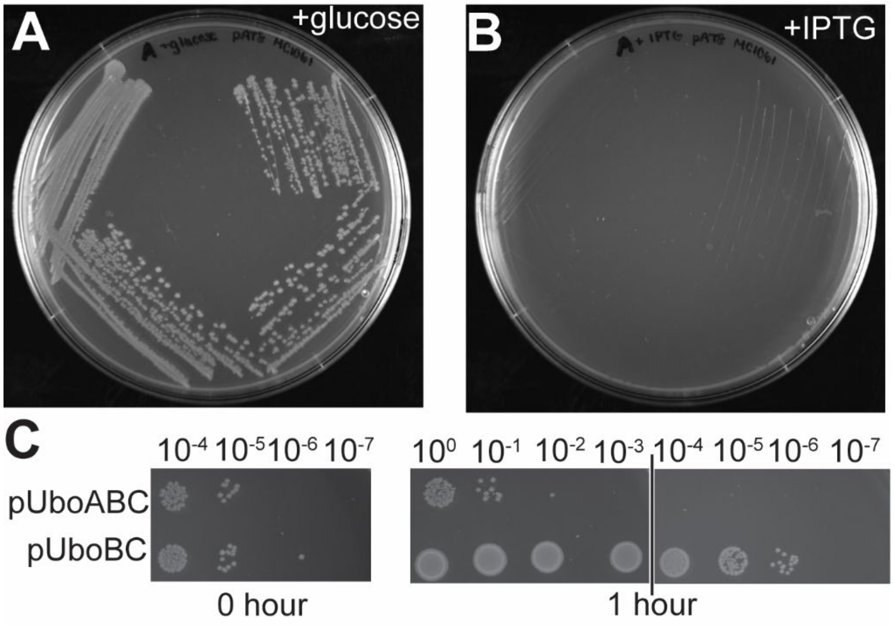

Figure S2. A: Streak of *E. coli* pUboABC onto an agar plate supplemented with 100 µg/mL ampicillin (to select for the plasmid) and 0.2% w/v glucose. This condition represses leaky expression of *uboA*, and colonies grew normally. **B**: Streak of *E. coli* pUboABC onto an agar plate supplemented with 100 µg/mL ampicillin and 1 mM IPTG. This condition induces expression of *uboA*, and no colonies grew. **C:** Spot dilution assay of *E. coli* pUboABC and *E. coli* pUboBC. Liquid cultures were plated immediately prior to induction (0 hour spots) and 1 hour after induction (1 hour spots). *E. coli* pUboABC had a clear growth deficiency 1 hour after induction compared to *E. coli* pUboBC.

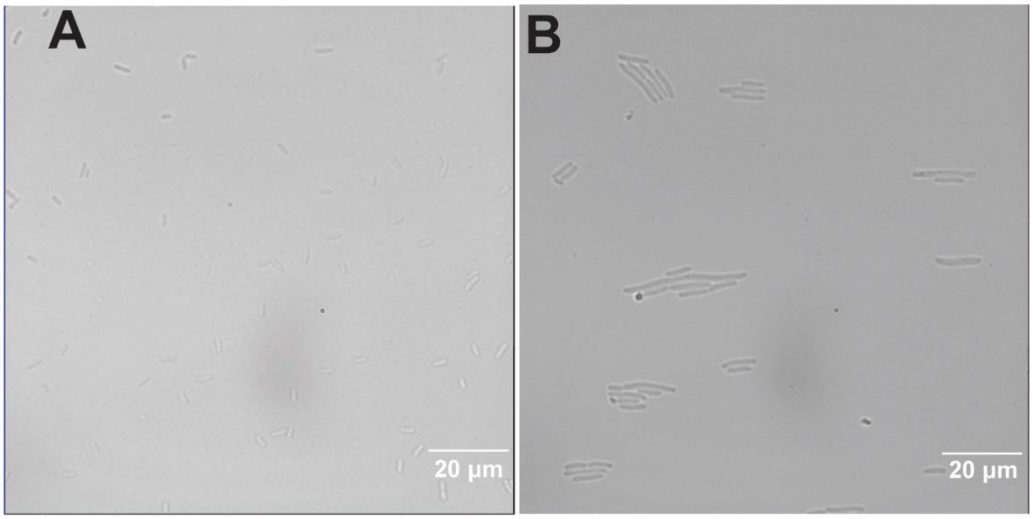

Figure S3. Microscopy on *E. coli* pUboBC and *E. coli* pUboABC. **A**: *E. coli* pUboBC cells had a normal rod shape two hours after induction with 100 µM IPTG. **B**: *E. coli* pUboABC cells two hours after induction with 100 µM IPTG tended to be longer than the cells depicted in panel A, indicative of filamentation.

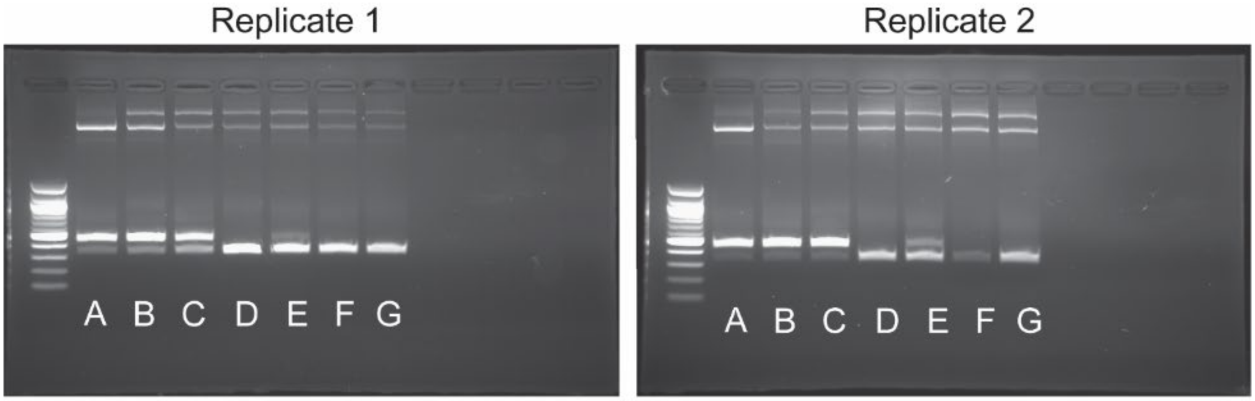

PCR	DNA Template
A	90% pUboABC, 10% pUboBC
B	Miniprepped DNA from transforming into *E. coli* MC1061
C	Miniprepped DNA from co-culture, immediately prior to IPTG induction
D	Miniprepped DNA from co-culture, 1 hour after 100 µM IPTG induction
E	Miniprepped DNA from co-culture, 1 hour after 10 µM IPTG induction
F	Miniprepped DNA from co-culture, 2 hours after 10 µM IPTG induction
G	Miniprepped DNA from co-culture, 3 hours after 10 µM IPTG induction

Figure S4. DNA that was PCR-amplified from co-cultures of *E. coli* pUboABC and *E. coli* pUboBC. The longer amplicon (447 bp band) corresponds to pUboABC and the shorter amplicon (336 bp band) corresponds to pUboBC. The co-culture primarily consisted of *E. coli* pUboABC until induction, after which the *E. coli* pUboBC cells began to overtake the co-culture.

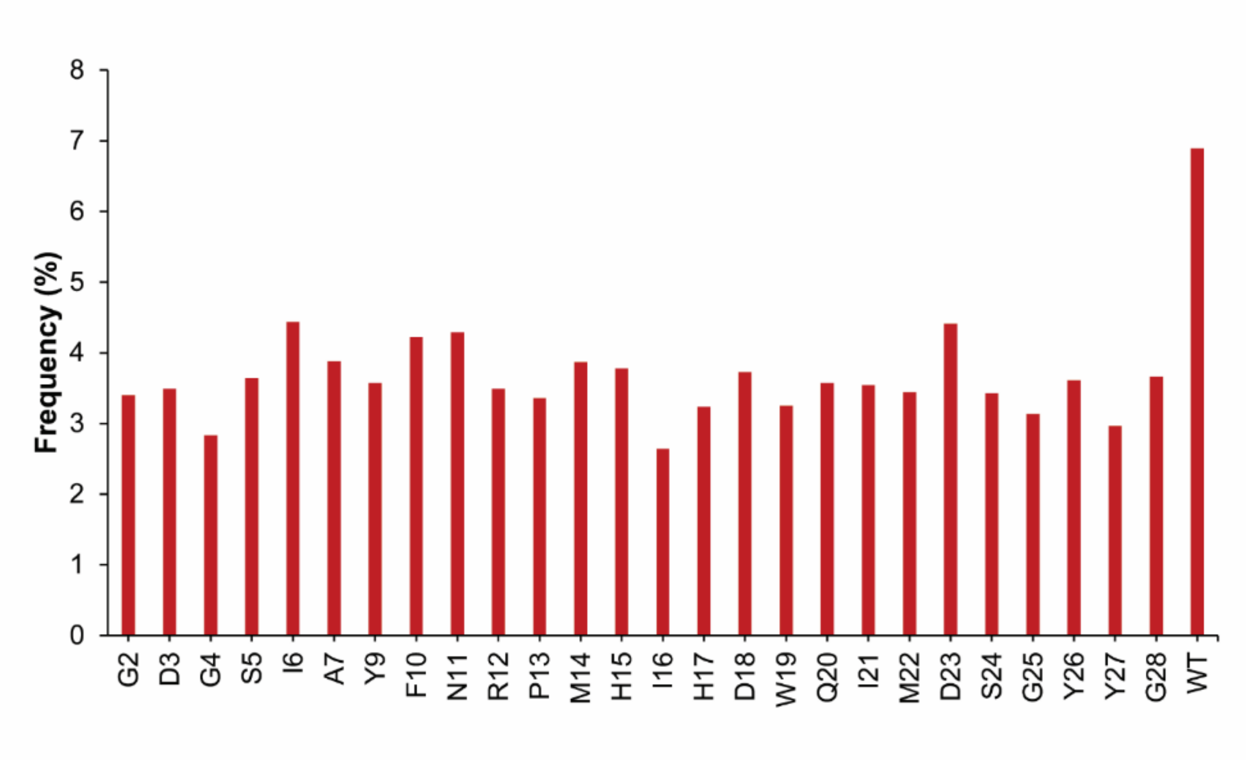

Figure S5. The frequencies of point variants in the cloning transformation library of the single mutant library were summed for each residue after sequencing on the NovaSeq (Equation S6), revealing that the residue-specific libraries were combined in roughly equimolar amounts. WT ubonodin was present at higher levels than expected, which could be due to the WT bias when conducting PCR with NNK codons, which has been documented previously.[4]

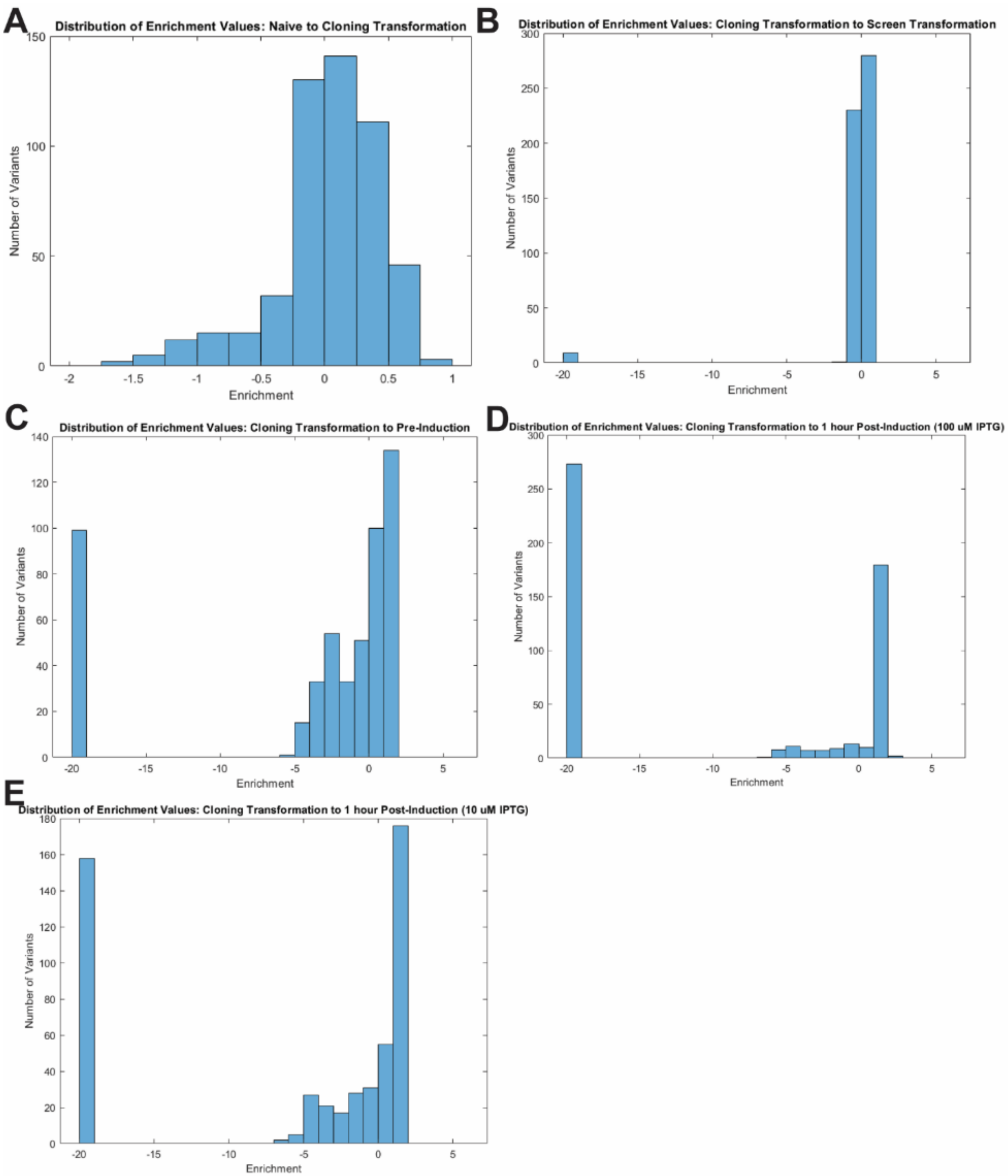

Figure S6. Histograms depicting the distribution of point variants' enrichment values at different steps of the single mutant screen (data from NovaSeq single mutant screen). Dropout variants were arbitrarily assigned an enrichment value of -20. Comparisons are between **A:** The naïve library and the cloning transformation library. Enrichment values have a small range, with 80% of variants falling between an enrichment value of -0.5 and 0.5. Only two variants (D3F and D3M) were not present in the cloning transformation library, likely because they had a low read count in the naïve library (626 reads and 554 reads respectively). **B:** The cloning transformation library and the screen transformation sample. **C**: The cloning transformation library and the pre-IPTG sample. **D**: The cloning transformation library and the 1-hour post-100 µM IPTG induction sample. **E:** The cloning transformation library and the 1-hour post-10 µM IPTG induction sample.

```
                 ring
MccJ25        GGAGHVP-EY-------FVGIGTPISFYG
Ubonodin      GGDGSIA-EYFNRPMHIHDWQIMDSGYYG
Citrocin      GGVGKII-EY---------FIGGGVGRYG
Cloacaenodin  GHSVDRIPEY-----FGPPGLPGPVLFYS
Acinetodin    GGKGPIF-ET----------WVTEGNYYG
Klebsidin     GSDGPII-EF---------FNPNGVMHYG
```

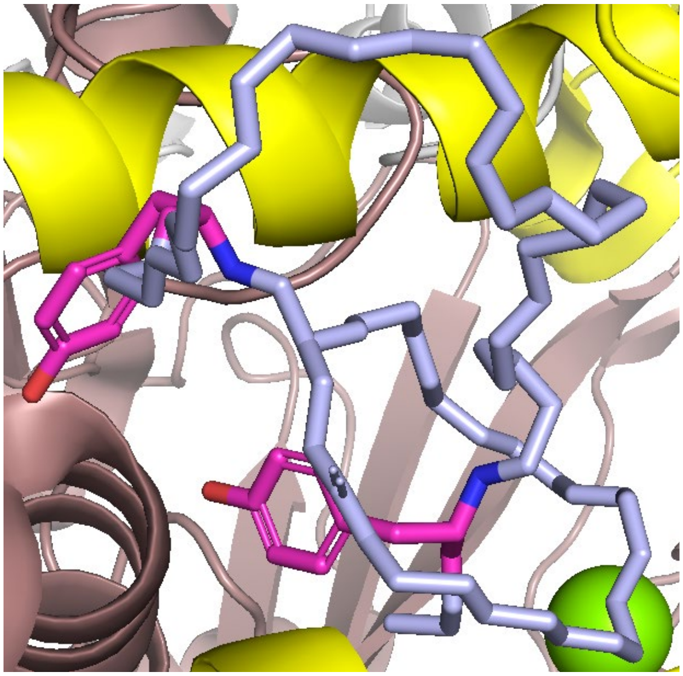

Figure S7. Conserved Tyr residues in antimicrobial lasso peptides make contacts with RNAP. Top: Alignment of several lasso peptides demonstrated or predicted to inhibit RNAP.[3, 5-7] The conserved Tyr residues in the position after the macrocyclic ring and in the penultimate position of the lasso peptide are highlighted in pink. All of the peptides have 8 aa rings except for cloacaenodin with a 9 aa ring. Note that acinetodin and klebsidin lack the conserved Tyr after the ring. Bottom: Crystal structure of MccJ25 bound to *E. coli* RNAP (PDB file 6N60). MccJ25 is in light blue with Tyr9 and Tyr20 in magenta. The β subunit of RNAP is in brown while the β' subunit is in yellow. The catalytic Mg^{+2} ion is also shown in green. The Tyr side chains of MccJ25 form multiple hydrogen bonds with the β and β' subunits.

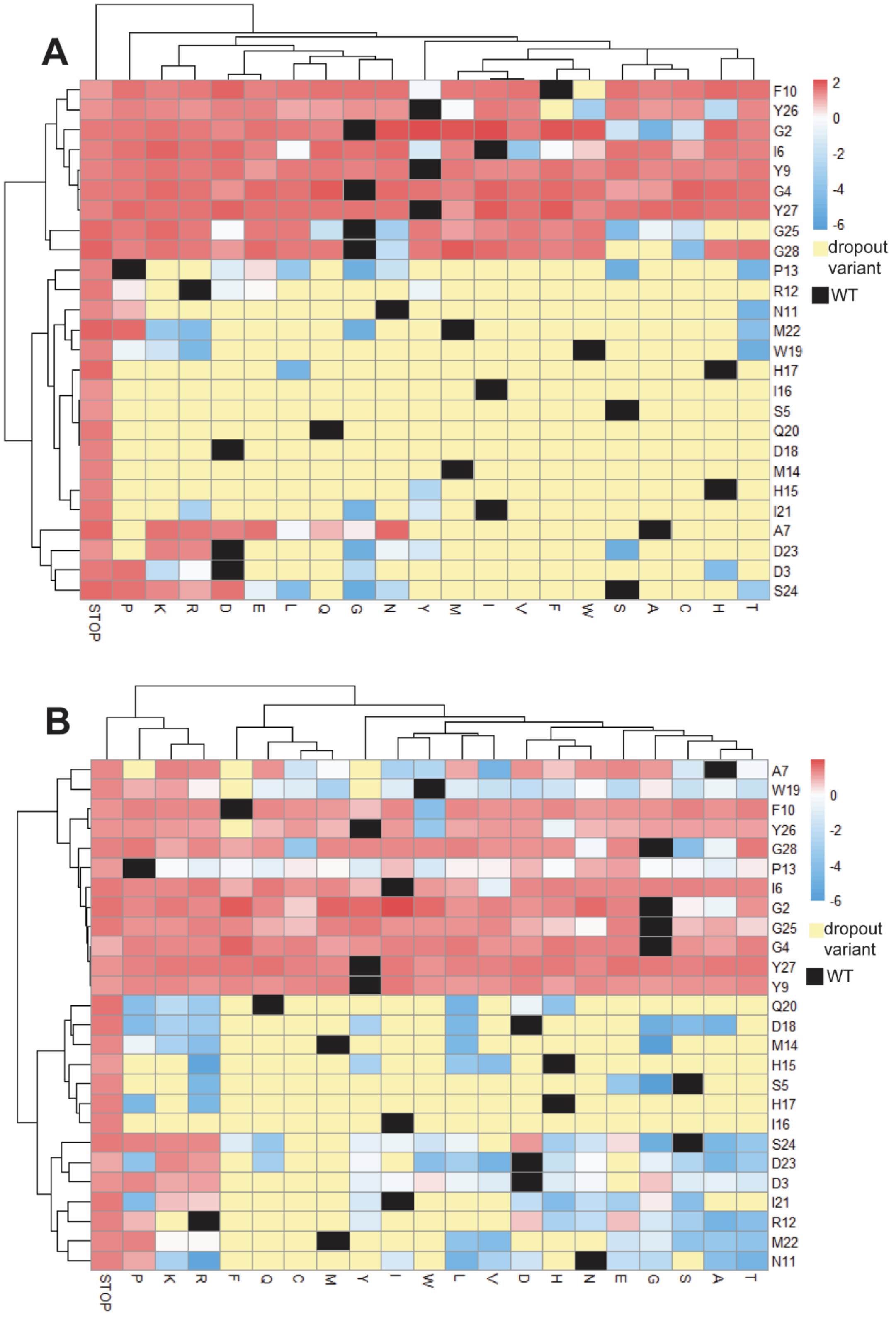

Figure S8. Hierarchical clustering of enrichment values for the single mutant library after induction with **A**: 100 µM IPTG or **B**: 10 µM IPTG. Data is obtained from the NovaSeq sequencing run of the single mutant library.

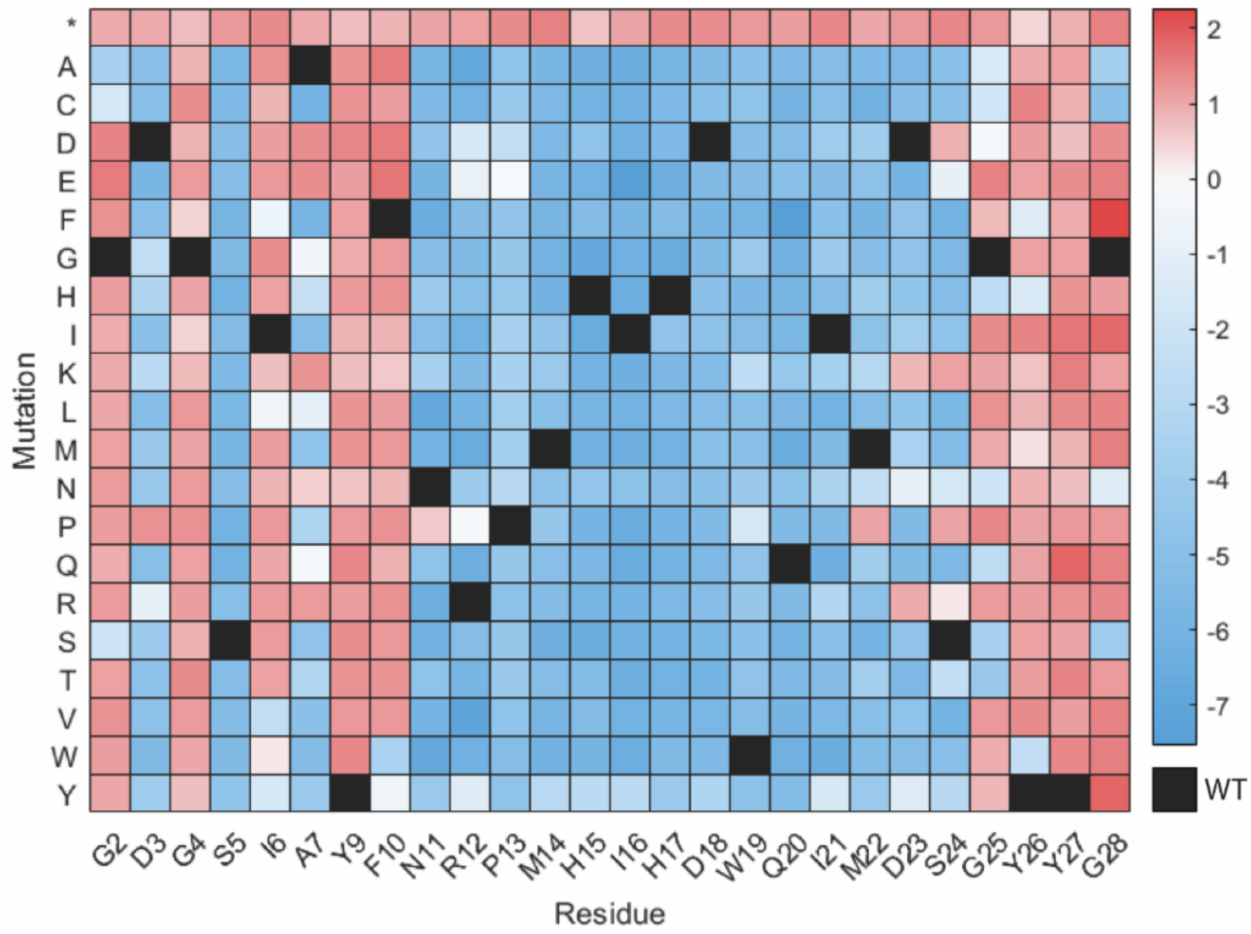

Figure S9. Enrichment values of the point variants present in the double mutant library (NovaSeq sequencing run on the double mutant library) after induction with 100 µM IPTG. The heatmap displays the same pattern of RNAP-inhibiting and non-RNAP inhibiting point variants as was observed in the single mutant library; compare to Figure 3A in the main text from the NovaSeq sequencing run on the single mutant library. There are no dropout single aa variants in the double mutant library, which is likely because we used more reads to sequence the double mutant library than the single mutant library.

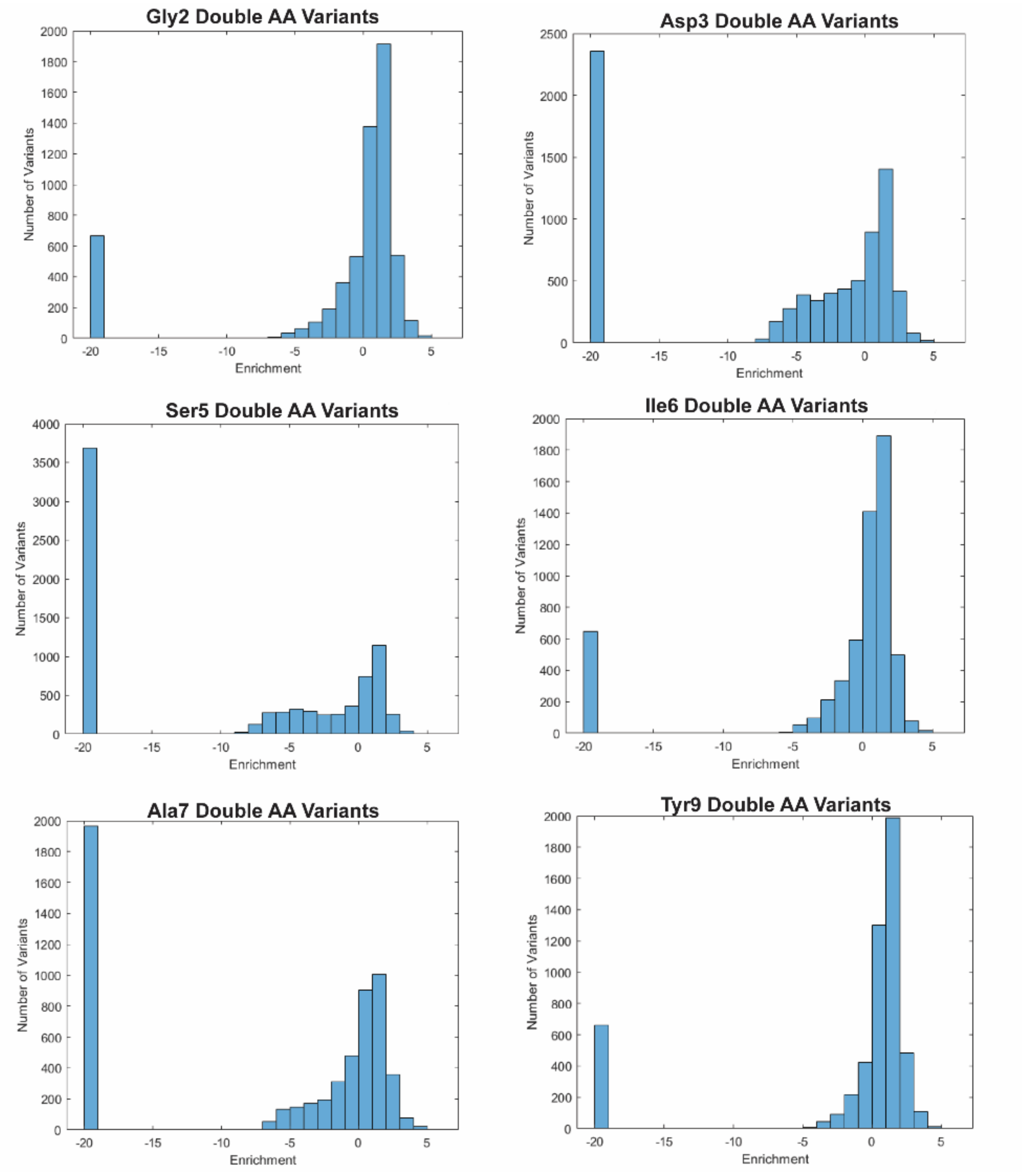
Gly2 Double AA Variants
Asp3 Double AA Variants
Ser5 Double AA Variants
Ile6 Double AA Variants
Ala7 Double AA Variants
Tyr9 Double AA Variants
Number of Variants
Enrichment

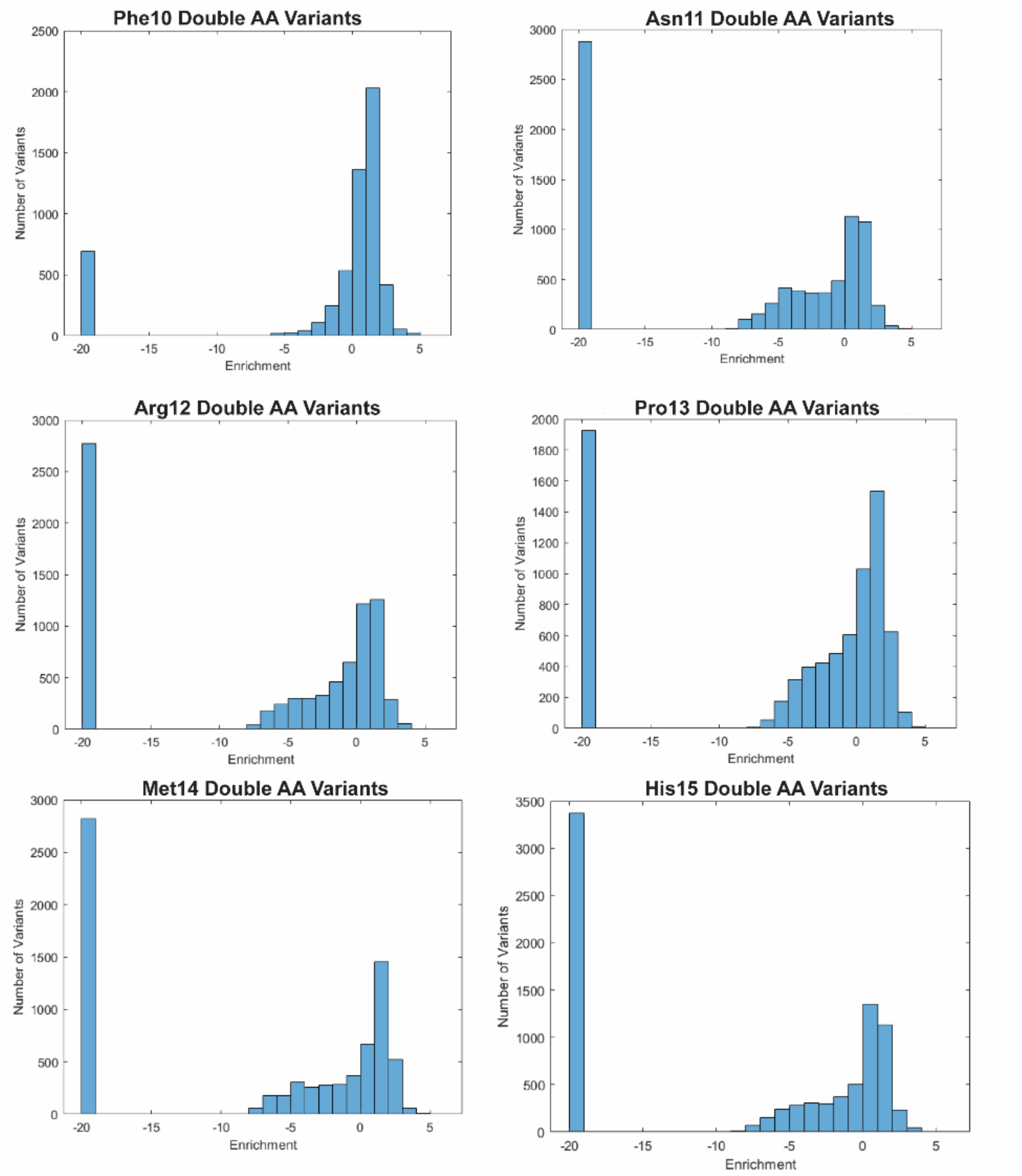
Phe10 Double AA Variants
2500
2000
1500
1000
500
0
Number of Variants
-20
-15
-10
-5
0
5
Enrichment
Asn11 Double AA Variants
3000
2500
2000
1500
1000
500
0
Number of Variants
-20
-15
-10
-5
0
5
Enrichment
Arg12 Double AA Variants
3000
2500
2000
1500
1000
500
0
Number of Variants
-20
-15
-10
-5
0
5
Enrichment
Pro13 Double AA Variants
2000
1800
1600
1400
1200
1000
800
600
400
200
0
Number of Variants
-20
-15
-10
-5
0
5
Enrichment
Met14 Double AA Variants
3000
2500
2000
1500
1000
500
0
Number of Variants
-20
-15
-10
-5
0
5
Enrichment
His15 Double AA Variants
3500
3000
2500
2000
1500
1000
500
0
Number of Variants
-20
-15
-10
-5
0
5
Enrichment

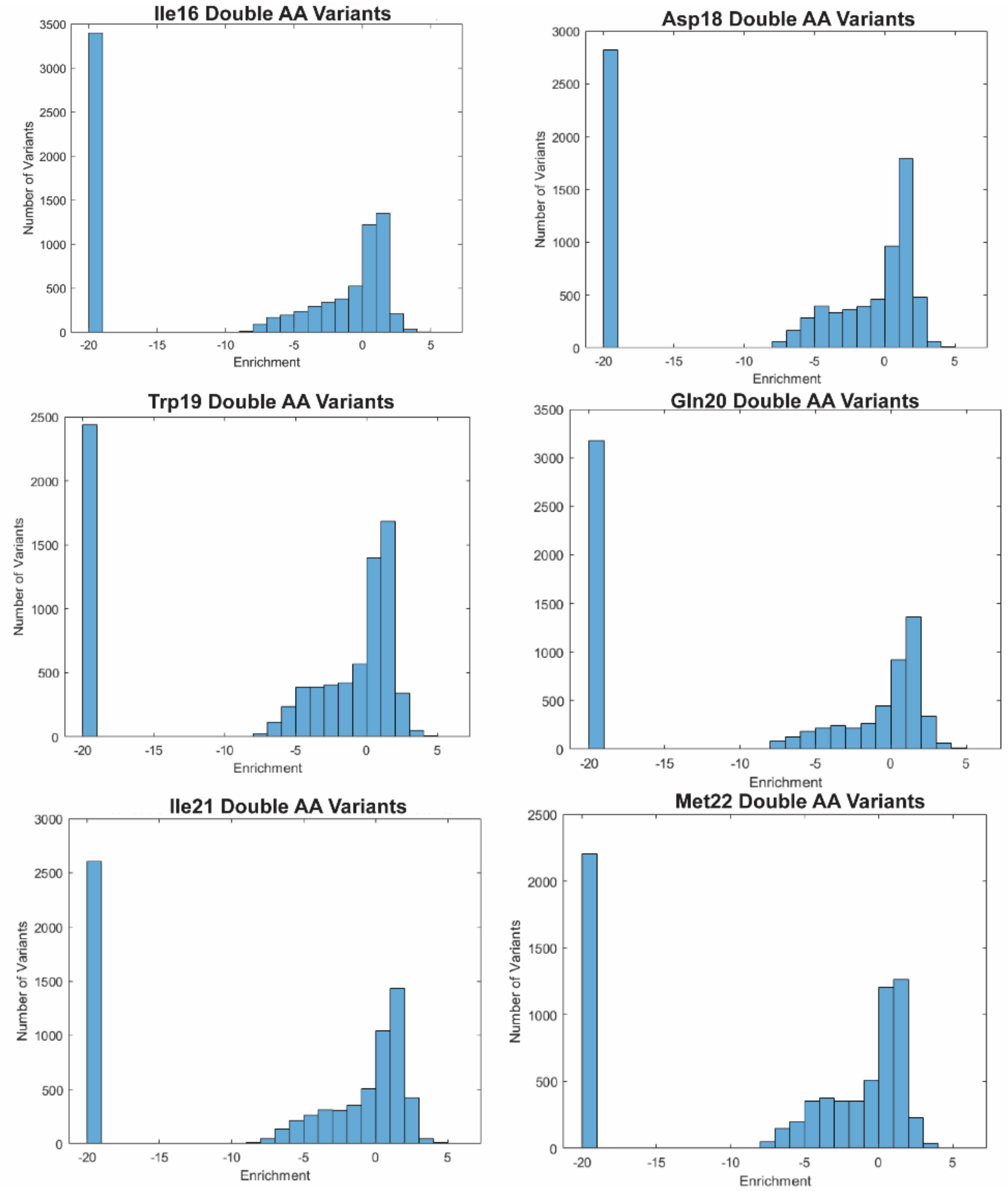
Ile16 Double AA Variants
Asp18 Double AA Variants
Trp19 Double AA Variants
Gln20 Double AA Variants
Ile21 Double AA Variants
Met22 Double AA Variants
Number of Variants
Enrichment
-20
-15
-10
-5
0
5
0
500
1000
1500
2000
2500
3000
3500

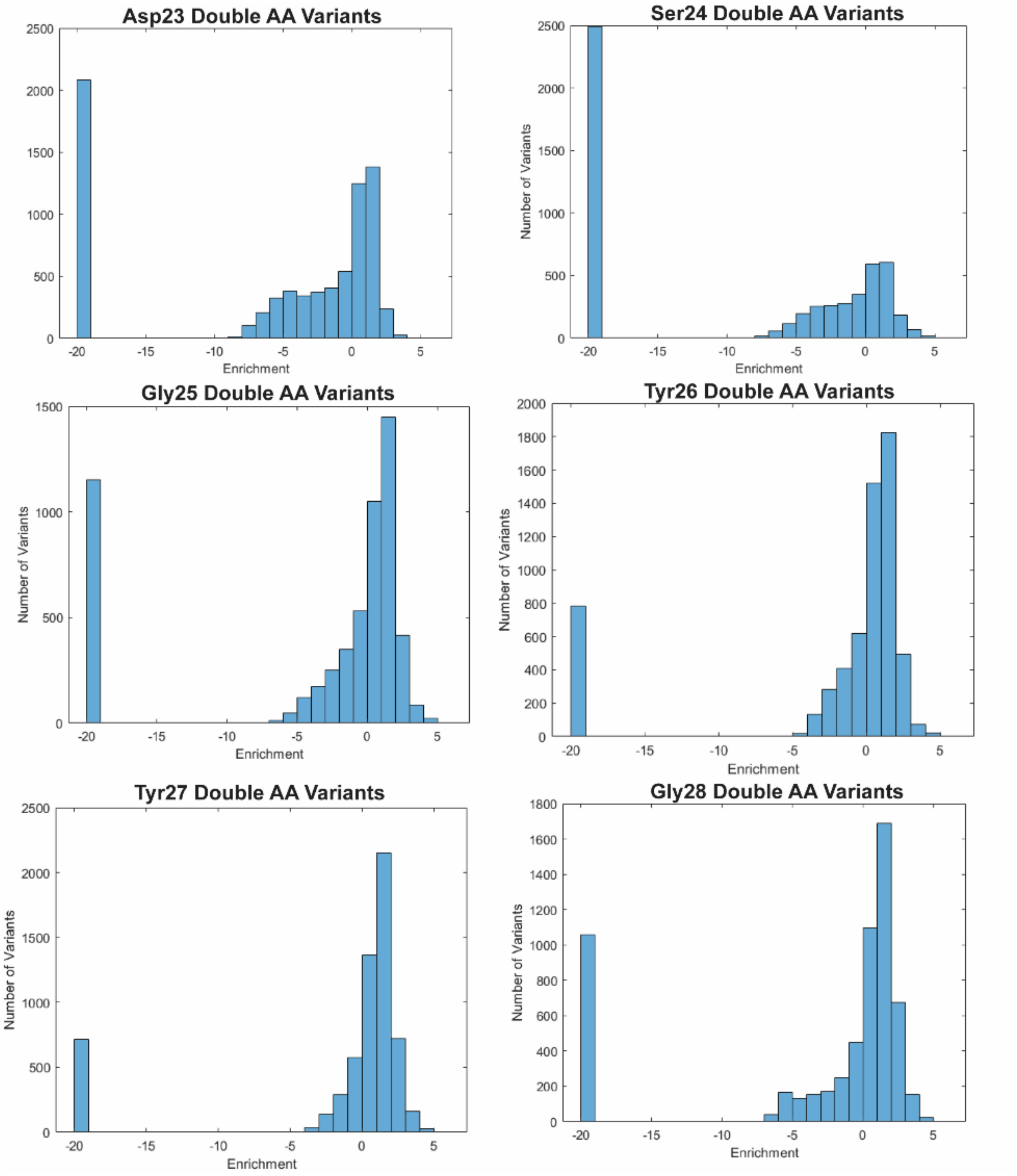

Figure S10. Histograms displaying the spread of enrichment values for each residue-specific subset of the double mutant library, excluding the Gly4 and His17 histograms (shown in Figure 5A-B) and positions that were not mutated. Dropout variants were arbitrarily assigned an enrichment value of -20.

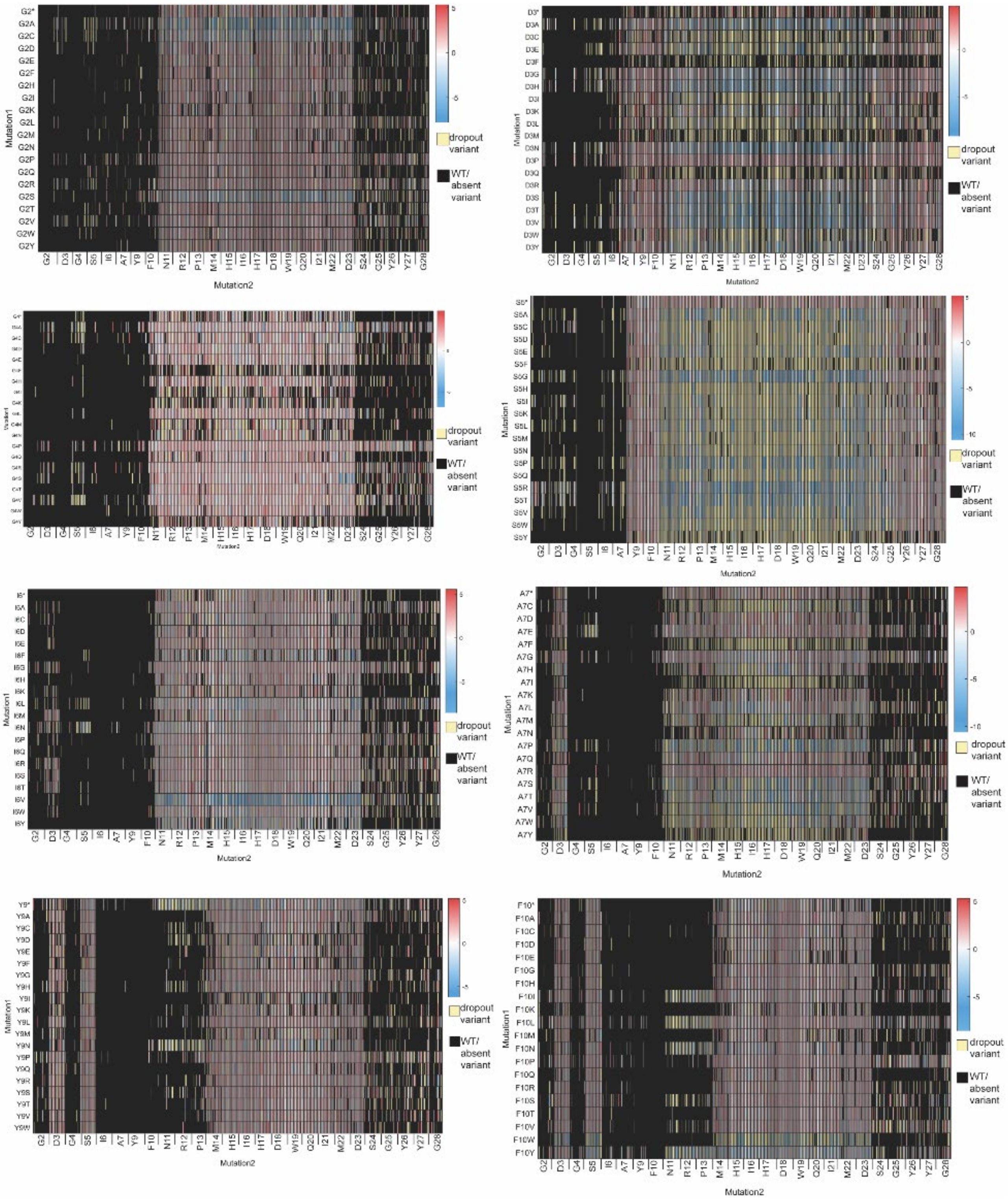
Mutation1
Mutation2
dropout variant
WT/ absent variant

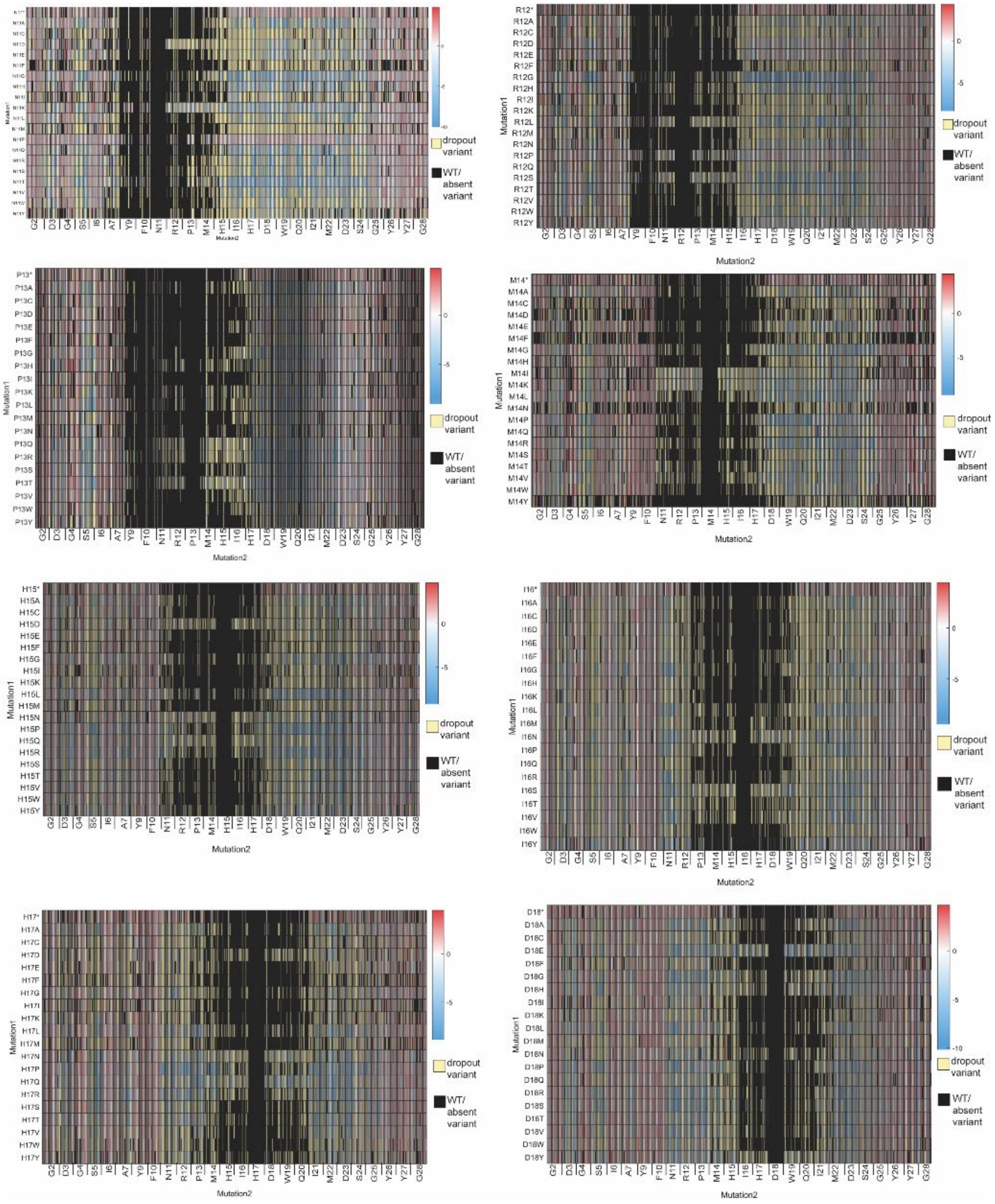
Mutation1
Mutation2
dropout variant
WT/ absent variant
0
-5
-10
G2 D3 G4 S5 I6 A7 Y9 F10 N11 R12 P13 M14 H15 I16 H17 D18 W19 Q20 I21 M22 D23 S24 G25 Y26 Y27 G28
R12* R12A R12C R12D R12E R12F R12G R12H R12I R12K R12L R12M R12N R12P R12Q R12S R12T R12V R12W R12Y
P13* P13A P13C P13D P13E P13F P13G P13H P13I P13K P13L P13M P13N P13Q P13R P13S P13T P13V P13W P13Y
M14* M14A M14C M14D M14E M14F M14G M14H M14I M14K M14L M14N M14P M14Q M14R M14S M14T M14V M14W M14Y
H15* H15A H15C H15D H15E H15F H15G H15I H15K H15L H15M H15N H15P H15Q H15R H15S H15T H15V H15W H15Y
I16* I16A I16C I16D I16E I16F I16G I16H I16K I16L I16M I16N I16P I16Q I16R I16S I16T I16V I16W I16Y
H17* H17A H17C H17D H17E H17F H17G H17I H17K H17L H17M H17N H17P H17Q H17R H17S H17T H17V H17W H17Y
D18* D18A D18C D18E D18F D18G D18H D18I D18K D18L D18M D18N D18P D18Q D18R D18S D18T D18V D18W D18Y

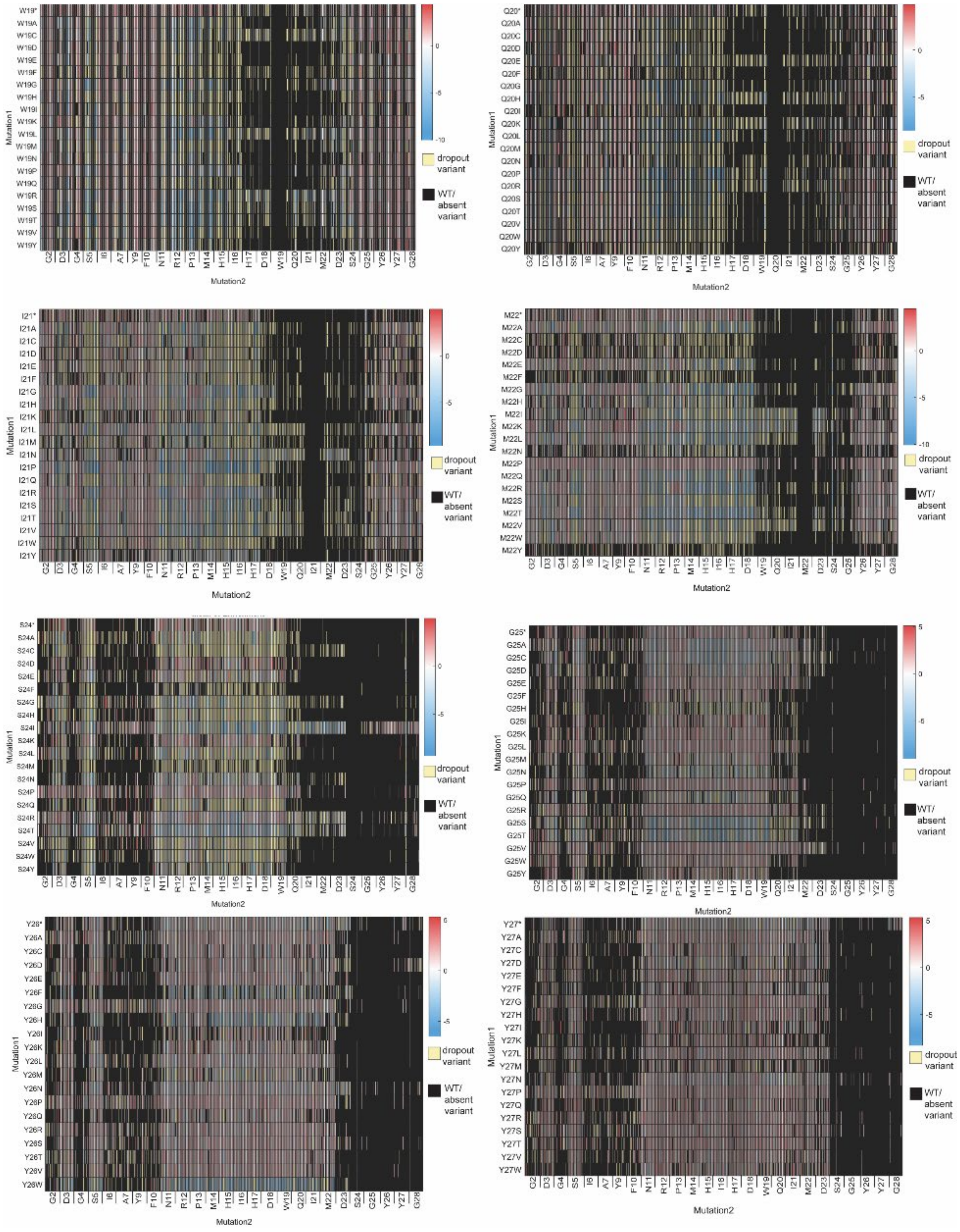
Mutation1
Mutation2
dropout variant
WT/ absent variant
0
-5
-10
5

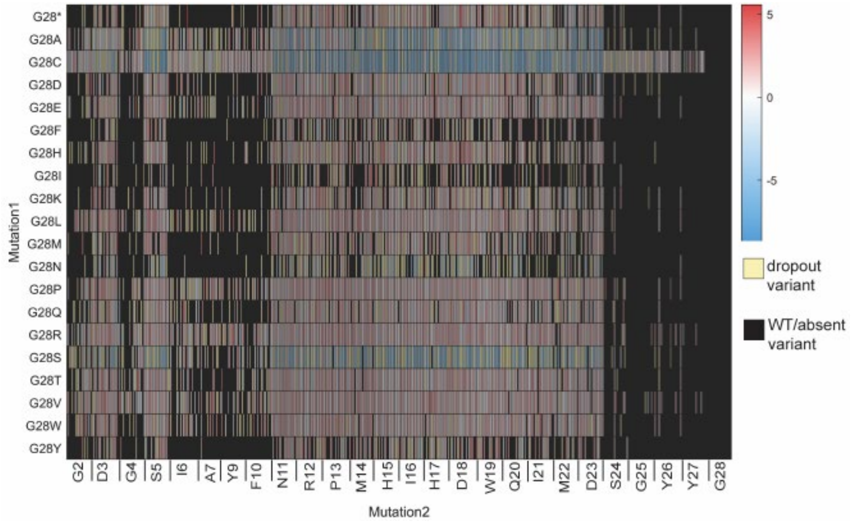

Figure S11. Non-clustered heatmaps of each residue-specific subset in the double mutant library.

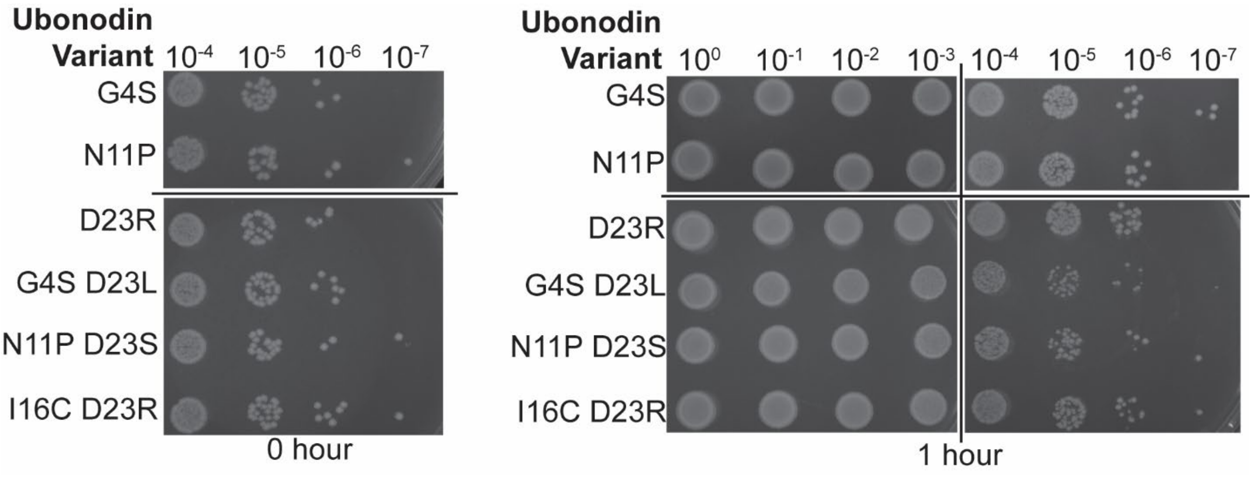

Figure S12. Spot dilution assay of the rescue variants and point variants without the compensatory mutation (in a pUboABC backbone). Liquid cultures were plated immediately prior to induction (0 hour spots) and 1 hour after induction (1 hour spots). The spots of point variants have healthy, normal-sized colonies 1 hour after induction whereas the spots of rescue variants have small, sick colonies 1 hour after induction. Biological duplicates of this assay were conducted, and this replicate is representative of the results from both replicates.

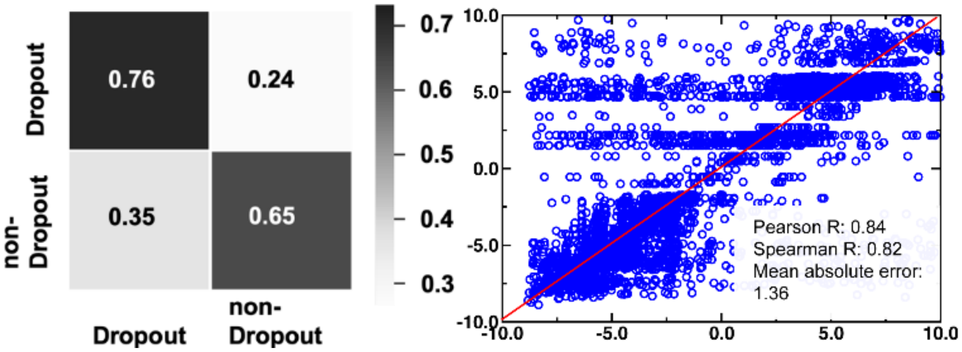

Figure S13. (*Left*) Confusion matrix for the classifier of DeepLasso in the training set. The matrix shows binary classification of dropout versus non-dropout variants with predicted outcomes on the x-axis and experimental observation on the y-axis. Grayscale is used to represent the magnitude of probability (i.e., high: black; low: white). (*Right*) Regression analysis for the regressor of DeepLasso in the training set. The linear correlation between experimental vs. predicted enrichment values is shown along with Pearson correlation coefficient, Spearman correlation coefficient, and mean absolute error.

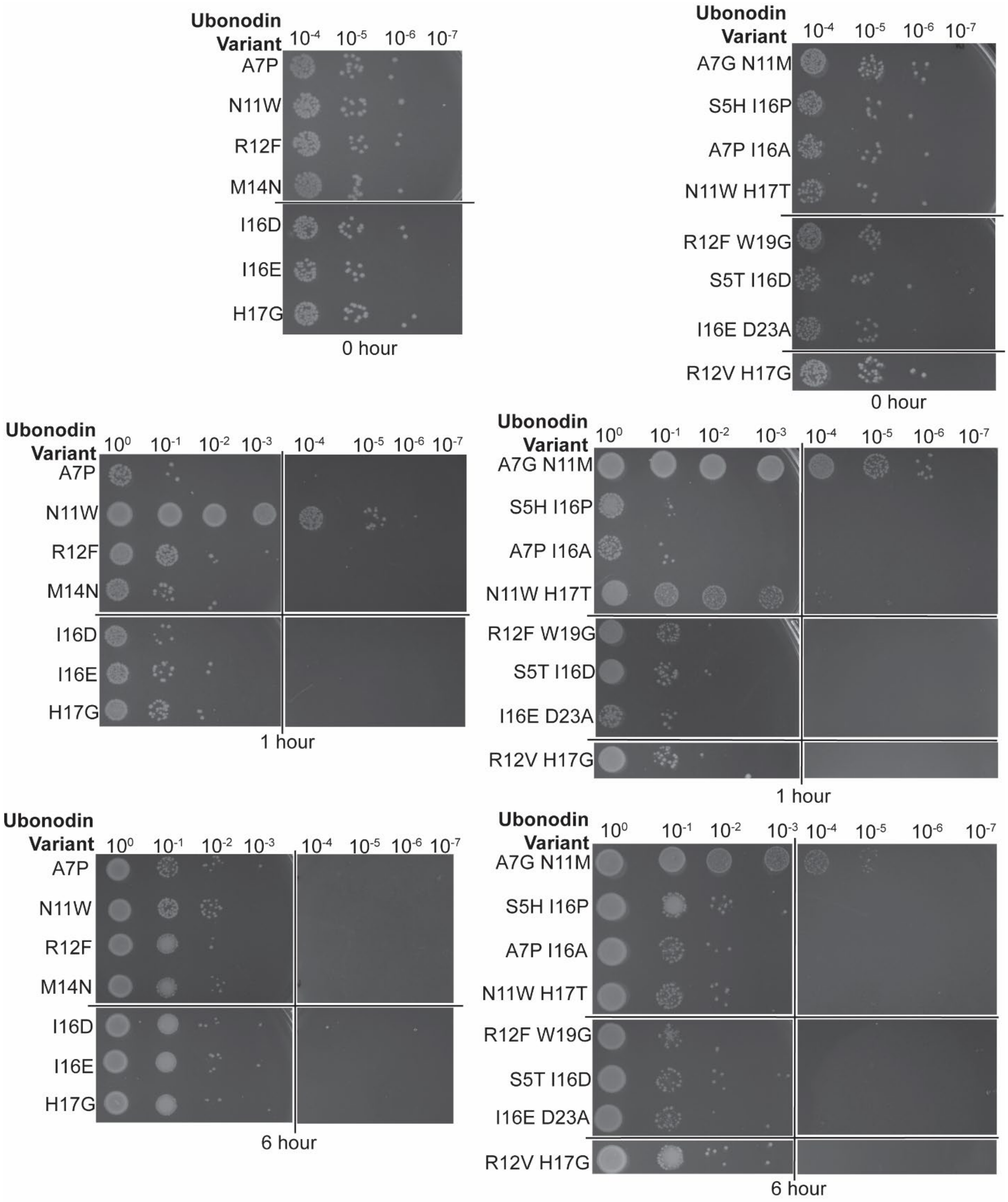

Figure S14. Spot dilution assays for the ubonodin variants (in a pUboABC backbone) identified from the screen with potential activity. The toxic effect of most variants was observed 1 hour after induction, though the toxic effect of ubonodin N11W and ubonodin A7G N11M was only observed 6 hours after induction. The apparent differences in toxicity could be because the screen incorporated information on leaky expression prior to induction, which cannot be done with the spot dilution assay. Furthermore, heterologous expression of ubonodin N11W in a pUboABCD vector demonstrated that the variant is expressed at very low levels, so it may take more time for ubonodin N11W to accumulate to levels that cause a visible difference in the spot dilution assay. Biological duplicates of this assay were conducted, and the replicate shown is representative of both duplicates.

Table S1. Enrichment values of rescue variants.

Single Amino Acid Variant	Single Amino Acid Variant Enrichment	Residue Implicated with Many Rescue Variants	Rescue Variant Enrichment	
**G4S**	1.1	Asp23	G4S D23A: -4.5<br>G4S D23C: -3.4<br>G4S D23E: -1.0<br>G4S D23F: dropout variant<br>G4S D23G: -2.2<br>G4S D23L: -4.926<br>G4S D23M: -3.2	G4S D23N: -0.1<br>G4S D23P: -2.9<br>G4S D23Q: -0.4<br>G4S D23S: -3.5<br>G4S D23V: -4.6<br>G4S D23W: -0.9
**N11P**	0.97	Asp23	N11P D23A: -5.1<br>N11P D23C: -5.6<br>N11P D23F: dropout variant<br>N11P D23G: -1.3<br>N11P D23I: dropout variant<br>N11P D23L: -4.6<br>N11P D23M: -5.0<br>N11P D23N: -0.3	N11P D23P: -5.4<br>N11P D23Q: -2.2<br>N11P D23S: -3.6<br>N11P D23T: -3.3<br>N11P D23V: -4.5<br>N11P D23W: -0.9<br>N11P D23Y: -1.1
**D23K**	1.2	Ile16	I16A D23K: -0.5<br>I16C D23K: -0.3<br>I16D D23K: -2.4<br>I16E D23K: -2.8<br>I16G D23K: -3.8<br>I16H D23K: -0.3<br>I16K D23K: -1.9	I16N D23K: -1.6<br>I16Q D23K: dropout variant<br>I16R D23K: -0.8<br>I16S D23K: -0.8<br>I16T D23K: -1.7<br>I16V D23K: -0.3<br>I16W D23K: -0.1
**D23R**	1.2	Ile16	I16A D23R: -1.7<br>I16C D23R: -2.9<br>I16D D23R: -3.2<br>I16E D23R: -1.9<br>I16G D23R: -3.1<br>I16H D23R: -1.9<br>I16K D23R: -2.5	I16M D23R: -0.4<br>I16N D23R: -1.6<br>I16P D23R: -0.1<br>I16Q D23R: -2.1<br>I16R D23R: -2.6<br>I16S D23R: -1.3<br>I16T D23R: -1.8
**Y26L**	1.1	Ile16	I16A Y26L: -0.4<br>I16C Y26L: -0.4<br>I16D Y26L: -1.0<br>I16E Y26L: -0.4<br>I16F Y26L: -0.1<br>I16G Y26L: -1.6<br>I16H Y26L: -0.2<br>I16K Y26L: -1.1	I16M Y26L: -0.3<br>I16N Y26L: -0.3<br>I16P Y26L: -0.5<br>I16Q Y26L: -1.5<br>I16R Y26L: -0.6<br>I16S Y26L: -0.9<br>I16T Y26L: -0.2

Table S2. Hyperparameters used in the encoders, classifier, and regressor of DeepLasso.

	Sequence encoder	Topology encoder	Classifier	Regressor
Convolutional Layers	3	3	1	0
LSTM Layers	2	0	0	0
Residual Blocks	0	0	2	1
MLP Layers	0	0	1	2
Output Channels	2048	768	N/A	N/A
Batch Size	512	512	64	64
Dropout	0.5	0.5	0.5	0.5
Learning Rate	1e-4	1e-4	2e-4	2e-4

Table S3. Statistics of types of variants involved in the training and test sets for classification and regression.

	Classifier training	Classifier testing	Regressor training	Regressor testing
Wild type	1	0	1	0
Single aa variant	370	8	222	19
Double aa variant	11940	997	6131	581
Triple aa variant	40875	3893	1639	174
Quadruple aa variant	2956	798	103	11
Higher-order aa variant	796	85	4	0
Stop codon	4745	387	2230	248

Table S4. Prediction results for single and double aa variants whose MICs were tested. The column variable, *Experimental observation*, refers to the enrichment for a lasso peptide variant observed in the screen. *DeepLasso prediction (classifier)* refers to the prediction outcome of DeepLasso in classifying a mutant to be dropout mutant or non-dropout variant. *DeepLasso prediction (regressor)* refers to enrichment value predicted for non-dropout variants. *Test or training (classifier)* and *Test or training (regressor)* refer to whether the tested variant appears in the training, testing set, or neither.

Variants	Experimental observation	DeepLasso prediction (classifier)	DeepLasso prediction (regressor)	Test or training (classifier)	Test or training (regressor)
A7G N11M	Dropout	Dropout	–	Testing	–
S5H I16P	Dropout	Dropout	–	Testing	–
N11W H17T	Dropout	Non-Dropout	-7.4	Training	–
R12V H17G	Dropout	Dropout	–	Training	–
I16E D23A	Dropout	Dropout	–	Training	–
S5T I16D	Dropout	Non-Dropout	-5.4	Testing	–
A7P I16A	Dropout	Non-Dropout	-5.3	Training	–
R12F W19G	Dropout	Dropout	–	Training	–
H17G	-6.5	Non-Dropout	-6.1	Training	Training
I16E	-7.5	Non-Dropout	-4.8	Training	Training
M14N	-4.7	Non-Dropout	-4.1	Training	Training
R12F	-5.3	Dropout	–	Training	Training
A7P	-3.4	Non-Dropout	-3.2	Training	Training
I16D	-6.2	Non-Dropout	-7.8	Training	Training
N11W	Dropout	Dropout	–	Training	–

Table S5. Molecular weights and MIC values (in μg/mL) of ubonodin variants.

Ubonodin Variant	Molecular Weight (g/mol)	*B. cenocepacia* AU0756 MIC (μg/mL)	*B. cenocepacia* AU24326 MIC (μg/mL)
**WT**	3217.49	0.8-1.6	6.4
**A7P**	3243.53	6.5	6.5 - 13
**R12F**	3208.48	6.4	26
**M14N**	3200.4	1.6 – 6.4	13
**I16D**	3219.42	1.6 – 6.4	26
**I16E**	3233.45	1.6 – 3.2	52
**H17G**	3137.4	0.39 – 0.78	6.3
**A7G N11M**	3220.56	>26	>52
**S5H I16P**	3251.51	26	>52
**A7P I16A**	3201.45	13 - 26	13 - 26
**R12F W19G**	3079.32	>25	>49
**S5T I16D**	3233.45	3.2 – 6.5	52
**I16E D23A**	3189.44	6.4 - 26	>51
**R12V H17G**	3080.35	0.77 – 1.5	12-25

Table S6. MIC values of various antibiotics against the *B. cenocepacia* clinical isolates used in this study.[8-10] WT ubonodin has a lower MIC than the other antibiotics reported in the literature. However, it is important to note that while we used the CLSI broth microdilution method to measure the MIC of ubonodin, the CLSI agar dilution method was used in the literature to measure the MIC of the other compounds. Two values were given for combination treatments: an MIC of 32/4 for ceftazidime-avibactam means that when the two antibiotics are used together, the bacteria must be treated with 32 μg/mL ceftazidime and 4 μg/mL avibactam to inhibit cell growth.

Compound	*B. cenocepacia* AU0756 MIC (μg/mL)	*B. cenocepacia* AU24326 MIC (μg/mL)
**WT ubonodin**	0.8 – 1.6	6.4
**Tobramycin**	512	512
**Ciprofloxacin**	256	8
**Minocycline**	8	32
**Imipenem**	128 - 256	128
**Ceftazidime**	128	32
**Trimethoprim-sulfamethoxazole**	8/152	4/76
**Ceftazidime-avibactam**	32/4	8/4
**Imipenem-relebactam**	32/4	Not reported
**Piperacillin-avibactam**	4/4	Not reported
**Piperacillin**	Not reported	512

Table S7. Primers used in this study.

Primer Name	Sequence
pQE-80 *Nhe*I For	GATCGCTAGCCATCAATTAAGA
pAT8 *Nco*I Rev	GCATCCATGGTCACTTCATGGTCTGGTTAGTTC
*Xho*I For Lib	AGCCGTCTTCACCTCGAG
*Nhe*I Rev Lib	TCTTAATTGATGGCTAGCTTG
G2 For Lib	GACAATGGGANNKGATGGCAGCA
G2 Rev Lib	TGCTGCCATCMNNTCCCATTGTC
D3 For Lib	GAGGCNNKGGCAGCAT
D3 Rev Lib	ATGCTGCCMNNGCCTC
G4 For Lib	GAGGCGATNNKAGCATTGC
G4 Rev Lib	GCAATGCTMNNATCGCCTC
S5 For Lib	AGGCGATGGCNNKATTGCGGAAT
S5 Rev Lib	ATTCCGCAATMNNGCCATCGCCT
I6 For Lib	GATGGCAGCNNKGCGGAATAC
I6 Rev Lib	GTATTCCGCMNNGCTGCCATC
A7 For Lib	CGATGGCAGCATTNNKGAATACTTTAACC
A7 Rev Lib	GGTTAAAGTATTCMNNAATGCTGCCATCG
Y9 For Lib	GCATTGCGGAANNKTTTAACCGTCC
Y9 Rev Lib	GGACGGTTAAAMNNTTCCGCAATGC
F10 For Lib	GAATACNNKAACCGTCCGA
F10 Rev Lib	TCGGACGGTTMNNGTATTC
N11 For Lib	CGGAATACTTTNNKCGTCCGATGCA
N11 Rev Lib	TGCATCGGACGMNNAAAGTATTCCG
R12 For Lib	GGAATACTTTAACNNKCCGATGCATATTC
R12 Rev Lib	GAATATGCATCGGMNNGTTAAAGTATTCC
P13 For Lib	GGAATACTTTAACCGTNNKATGCATATTCATGATT
P13 Rev Lib	AATCATGAATATGCATMNNACGGTTAAAGTATTCC
M14 For Lib	TTTAACCGTCCGNNKCATATTCATGA
M14 Rev Lib	TCATGAATATGMNNCGGACGGTTAAA
H15 For Lib	ACCGTCCGATGNNKATTCATGAT
H15 Rev Lib	ATCATGAATMNNCATCGGACGGT
I16 For Lib	GTCCGATGCATNNKCATGATTGGCA
I16 Rev Lib	TGCCAATCATGMNNATGCATCGGAC
H17 For Lib	CGATGCATATTNNKGATTGGCAGAT
H17 Rev Lib	ATCTGCCAATCMNNAATATGCATCG
D18 For Lib	GATGCATATTCATNNKTGGCAGATTATGG
D18 Rev Lib	CCATAATCTGCCAMNNATGAATATGCATC
W19 For Lib	ATGCATATTCATGATNNKCAGATTATGGATAGC
W19 Rev Lib	GCTATCCATAATCTGMNNATCATGAATATGCAT
Q20 For Lib	ATATTCATGATTGGNNKATTATGGATAGCGG
Q20 Rev Lib	CCGCTATCCATAATMNNCCAATCATGAATAT
I21 For Lib	GATTGGCAGNNKATGGATAGCG

I21 Rev Lib	CGCTATCCATMNNCTGCCAATC
M22 For Lib	TGGCAGATTNNKGATAGCGGC
M22 Rev Lib	GCCGCTATCMNNAATCTGCCA
D23 For Lib	GGCAGATTATGNNKAGCGGCTATTA
D23 Rev Lib	TAATAGCCGCTMNNCATAATCTGCC
S24 For Lib	CAGATTATGGATNNKGGCTATTATGGC
S24 Rev Lib	GCCATAATAGCCMNNATCCATAATCTG
G25 For Lib	AGATTATGGATAGCNNKTATTATGGCTGAAA
G25 Rev Lib	TTTCAGCCATAATAMNNGCTATCCATAATCT
Y26 For Lib	GGATAGCGGCNNKTATGGCTGAA
Y26 Rev Lib	TTCAGCCATAMNNGCCGCTATCC
Y27 For Lib	ATAGCGGCTATNNKGGCTGAAAGCT
Y27 Rev Lib	AGCTTTCAGCCMNNATAGCCGCTAT
G28 For Lib	TAGCGGCTATTATNNKTGAAAGCTTAATT
G28 Rev Lib	AATTAAGCTTTCAMNNATAATAGCCGCTA
pAT8 G2F Lib For	CAATGGGATTTGATGGCAGC
pAT8 G2F Lib Rev	GCTGCCATCAAATCCCATTG
pAT8 G2I Lib For	CAATGGGAATTGATGGCAGC
pAT8 G2I Lib Rev	GCTGCCATCAATTCCCATTG
pAT8 G2M Lib For	CAATGGGAATGGATGGCAGC
pAT8 G2M Lib Rev	GCTGCCATCCATTCCCATTG
pAT8 G2N Lib For	CAATGGGAAATGATGGCAGC
pAT8 G2N Lib Rev	GCTGCCATCATTTCCCATTG
pAT8 G2W Lib For	CAATGGGATGGGATGGCAGC
pAT8 G2W Lib Rev	GCTGCCATCCCATCCCATTG
pAT8 G2Y Lib For	CAATGGGATATGATGGCAGC
pAT8 G2Y Lib Rev	GCTGCCATCATATCCCATTG
pAT8 A7P For	ATGGCAGCATTCCGGAATACTTTAA
pAT8 A7P Rev	TTAAAGTATTCCGGAATGCTGCCAT
pAT8 N11W For	GGAATACTTTTGGCGTCCGATGC
pAT8 N11W Rev	GCATCGGACGCCAAAAGTATTCC
pAT8 R12F For	GGAATACTTTAACTTTCCGATGCATATTC
pAT8 R12F Rev	GAATATGCATCGGAAAGTTAAAGTATTCC
pAT8 M14N For	CTTTAACCGTCCGAATCATATTCATGA
pAT8 M14N Rev	TCATGAATATGATTCGGACGGTTAAAG
pAT8 I16D For	GTCCGATGCATGATCATGATTGG
pAT8 I16D Rev	CCAATCATGATCATGCATCGGAC
pAT8 I16E For	GTCCGATGCATGAGCATGATTGG
pAT8 I16E Rev	CCAATCATGCTCATGCATCGGAC
pAT8 H17G For	CGATGCATATTGGTGATTGGCAGA
pAT8 H17G Rev	TCTGCCAATCACCAATATGCATCG
A7G N11M For	CATTGGTGAATACTTTATGCGTCCGATG
A7G N11M Rev	CATCGGACGCATAAAGTATTCACCAATG

S5H I16P For v2	GATGGCCATATTGCGGAATACTTTAACCGTCCGATGCATCCGCA TGATTG
S5H I16P Rev v2	CAATCATGCGGATGCATCGGACGGTTAAAGTATTCCGCAATATG GCCATC
N11W H17T For	CGATGCATATTACCGATTGGCAGAT
N11W H17T Rev	ATCTGCCAATCGGTAATATGCATCG
A7P I16A For	CGATGCATGCGCATGATTGGCA
A7P I16A Rev	TGCCAATCATGCGCATGCATCG
R12F W19G For	CATATTCATGATGGTCAGATTATGGATAGCG
R12F W19G Rev	CGCTATCCATAATCTGACCATCATGAATATG
S5T I16D For	GATGGCACCATTGCGGA
S5T I16D Rev	TCCGCAATGGTGCCATC
I16E D23A For	CAGATTATGGCGAGCGGCTATT
I16E D23A Rev	AATAGCCGCTCGCCATAATCTG
R12V H17G For	GGAATACTTTAACGTGCCGATGCATATT
R12V H17G Rev	AATATGCATCGGCACGTTAAAGTATTCC
pAT8 G4S For	AGGCGATAGCAGCATT
pAT8 G4S Rev	AATGCTGCTATCGCCT
pAT8 N11P For	GAATACTTTCCGCGTCCGATGCA
pAT8 N11P Rev	TGCATCGGACGCGGAAAGTATTC
pAT8 D23R For	GCAGATTATGCGTAGCGGCTATT
pAT8 D23R Rev	AATAGCCGCTACGCATAATCTGC
pAT8 D23L For	GCAGATTATGCTGAGCGGCTATT
pAT8 D23L Rev	AATAGCCGCTCAGCATAATCTGC
pAT8 D23S For	GCAGATTATGAGCAGCGGCTATT
pAT8 D23S Rev	AATAGCCGCTGCTCATAATCTGC
pAT8 I16C For	CGATGCATTGCCATGATTGGC
pAT8 I16C Rev	GCCAATCATGGCAATGCATCG
pQE-80 *Eco*RI For	GTGAGCGGATAACAATTTCACACAGAATTCATTAAAGAGG
pQE-80 *Hin*dIII Rev	GGATCTATCAACAGGAGTCCAAGCTCAGCTAATTAAG
P5-AT1F	AATGATACGGCGACCACCGAGATCTACACCGTGCGACAATGGG A
P7-AT1R1	CAAGCAGAAGACGGCATACGAGATCGTGATGCTCAGCTAATTAA GCTTTCA
P7-AT1R2	CAAGCAGAAGACGGCATACGAGATACATCGGCTCAGCTAATTAA GCTTTCA
P7-AT1R3	CAAGCAGAAGACGGCATACGAGATGCCTAAGCTCAGCTAATTAA GCTTTCA
P7-AT1R4	CAAGCAGAAGACGGCATACGAGATTGGTCAGCTCAGCTAATTAA GCTTTCA
P7-AT1R5	CAAGCAGAAGACGGCATACGAGATCACTGTGCTCAGCTAATTAA GCTTTCA
P7-AT1R6	CAAGCAGAAGACGGCATACGAGATATTGGCGCTCAGCTAATTAA GCTTTCA
P7-AT1R7	CAAGCAGAAGACGGCATACGAGATGATCTGGCTCAGCTAATTAA GCTTTCA

P7-AT1R8	CAAGCAGAAGACGGCATACGAGATTCAAGTGCTCAGCTAATTAA GCTTTCA
P7-AT1R9	CAAGCAGAAGACGGCATACGAGATCTGATCGCTCAGCTAATTAA GCTTTCA
P7-AT1R10	CAAGCAGAAGACGGCATACGAGATAAGCTAGCTCAGCTAATTAA GCTTTCA
P7-AT1R11	CAAGCAGAAGACGGCATACGAGATGTAGCCGCTCAGCTAATTAA GCTTTCA
P7-AT1R12	CAAGCAGAAGACGGCATACGAGATTACAAGGCTCAGCTAATTAA GCTTTCA
P7-AT1R13	CAAGCAGAAGACGGCATACGAGATTTGACTGCTCAGCTAATTAA GCTTTCA
P7-AT1R14	CAAGCAGAAGACGGCATACGAGATGGAACTGCTCAGCTAATTAA GCTTTCA
P7-AT1R15	CAAGCAGAAGACGGCATACGAGATTGACATGCTCAGCTAATTAA GCTTTCA
P7-AT1R16	CAAGCAGAAGACGGCATACGAGATGGACGGGCTCAGCTAATTA AGCTTTCA

Table S8. Plasmids used in this study.

Plasmid Name	Template for PCR	Mutagenic Forward Primer for Cloning	Mutagenic Reverse Primer for Cloning	Vector backbone
pWC99	N/A	N/A	N/A	N/A
pUboABC	pWC99	N/A	N/A	pWC99
pUboBC	N/A	N/A	N/A	pQE-80
pUboABC G2F	pWC99	pAT8 G2F Lib For	pAT8 G2F Lib Rev	pUboABC
pUboABC G2I	pWC99	pAT8 G2I Lib For	pAT8 G2I Lib Rev	pUboABC
pUboABC G2M	pWC99	pAT8 G2M Lib For	pAT8 G2M Lib Rev	pUboABC
pUboABC G2N	pWC99	pAT8 G2N Lib For	pAT8 G2N Lib Rev	pUboABC
pUboABC G2W	pWC99	pAT8 G2W Lib For	pAT8 G2W Lib Rev	pUboABC
pUboABC G2Y	pWC99	pAT8 G2Y Lib For	pAT8 G2Y Lib Rev	pUboABC
pUboABC A7P	pWC99	pAT8 A7P For	pAT8 A7P Rev	pUboABC
pUboABC N11W	pWC99	pAT8 N11W For	pAT8 N11W Rev	pUboABC
pUboABC R12F	pWC99	pAT8 R12F For	pAT8 R12F Rev	pUboABC
pUboABC M14N	pWC99	pAT8 M14N For	pAT8 M14N Rev	pUboABC
pUboABC I16D	pWC99	pAT8 I16D For	pAT8 I16D Rev	pUboABC
pUboABC I16E	pWC99	pAT8 I16E For	pAT8 I16E Rev	pUboABC
pUboABC H17G	pWC99	pAT8 H17G For	pAT8 H17G Rev	pUboABC
pUboABCD A7P	pWC99	pAT8 A7P For	pAT8 A7P Rev	pWC99
pUboABCD N11W	pWC99	pAT8 N11W For	pAT8 N11W Rev	pWC99
pUboABCD R12F	pWC99	pAT8 R12F For	pAT8 R12F Rev	pWC99
pUboABCD M14N	pWC99	pAT8 M14N For	pAT8 M14N Rev	pWC99
pUboABCD I16D	pWC99	pAT8 I16D For	pAT8 I16D Rev	pWC99
pUboABCD I16E	pWC99	pAT8 I16E For	pAT8 I16E Rev	pWC99
pUboABCD H17G	pWC99	pAT8 H17G For	pAT8 H17G Rev	pWC99
pUboABC A7G N11M	pWC99	A7G N11M For	A7G N11M Rev	pUboABC
pUboABC S5H I16P	pWC99	S5H I16P For v2	S5H I16P Rev v2	pUboABC
pUboABC A7P I16A	pUboABCD A7P	A7P I16A For	A7P I16A Rev	pUboABC
pUboABC N11W H17T	pUboABCD N11W	N11W H17T For	N11W H17T Rev	pUboABC
pUboABC R12F W19G	pUboABCD R12F	R12F W19G For	R12F W19G Rev	pUboABC
pUboABC S5T I16D	pUboABCD I16D	S5T I16D For	S5T I16D Rev	pUboABC

pUboABC I16E D23A	pUboABCD I16E	I16E D23A For	I16E D23A Rev	pUboABC
pUboABC R12V H17G	pUboABCD H17G	R12V H17G For	R12V H17G Rev	pUboABC
pUboABCD A7G N11M	pWC99	A7G N11M For	A7G N11M Rev	pWC99
pUboABCD S5H I16P	pWC99	S5H I16P For v2	S5H I16P Rev v2	pWC99
pUboABCD A7P I16A	pUboABCD A7P	A7P I16A For	A7P I16A Rev	pWC99
pUboABCD N11W H17T	pUboABCD N11W	N11W H17T For	N11W H17T Rev	pWC99
pUboABCD R12F W19G	pUboABCD R12F	R12F W19G For	R12F W19G Rev	pWC99
pUboABCD S5T I16D	pUboABCD I16D	S5T I16D For	S5T I16D Rev	pWC99
pUboABCD I16E D23A	pUboABCD I16E	I16E D23A For	I16E D23A Rev	pWC99
pUboABCD R12V H17G	pUboABCD H17G	R12V H17G For	R12V H17G Rev	pWC99
pUboABC G4S	pWC99	pAT8 G4S For	pAT8 G4S Rev	pUboABC
pUboABC N11P	pWC99	pAT8 N11P For	pAT8 N11P Rev	pUboABC
pUboABC D23R	pWC99	pAT8 D23R For	pAT8 D23R Rev	pUboABC
pUboABC G4S D23L	pUboABC G4S	pAT8 D23L For	pAT8 D23L Rev	pUboABC
pUboABC N11P D23S	pUboABC N11P	pAT8 D23S For	pAT8 D23S Rev	pUboABC
pUboABC I16C D23R	pUboABC D23R	pAT8 I16C For	pAT8 I16C Rev	pUboABC

Table S9. Mutagenic primers used for constructing residue-specific libraries.

Residue-Specific Library	Mutagenic Forward Primer	Mutagenic Reverse Primer
G2 Single Mutant	G2 For Lib	G2 Rev Lib
D3 Single Mutant	D3 For Lib	D3 Rev Lib
G4 Single Mutant	G4 For Lib	G4 Rev Lib
S5 Single Mutant	S5 For Lib	S5 Rev Lib
I6 Single Mutant	I6 For Lib	I6 Rev Lib
A7 Single Mutant	A7 For Lib	A7 Rev Lib
Y9 Single Mutant	Y9 For Lib	Y9 Rev Lib
F10 Single Mutant	F10 For Lib	F10 Rev Lib
N11 Single Mutant	N11 For Lib	N11 Rev Lib
R12 Single Mutant	R12 For Lib	R12 Rev Lib
P13 Single Mutant	P13 For Lib	P13 Rev Lib
M14 Single Mutant	M14 For Lib	M14 Rev Lib
H15 Single Mutant	H15 For Lib	H15 Rev Lib
I16 Single Mutant	I16 For Lib	I16 Rev Lib
H17 Single Mutant	H17 For Lib	H17 Rev Lib
D18 Single Mutant	D18 For Lib	D18 Rev Lib
W19 Single Mutant	W19 For Lib	W19 Rev Lib
Q20 Single Mutant	Q20 For Lib	Q20 Rev Lib
I21 Single Mutant	I21 For Lib	I21 Rev Lib
M22 Single Mutant	M22 For Lib	M22 Rev Lib
D23 Single Mutant	D23 For Lib	D23 Rev Lib
S24 Single Mutant	S24 For Lib	S24 Rev Lib
G25 Single Mutant	G25 For Lib	G25 Rev Lib
Y26 Single Mutant	Y26 For Lib	Y26 Rev Lib
Y27 Single Mutant	Y27 For Lib	Y27 Rev Lib
G28 Single Mutant	G28 For Lib	G28 Rev Lib
D3 Double Mutant	D3 For Lib	D3 Rev Lib
S5 Double Mutant	S5 For Lib	S5 Rev Lib
N11 Double Mutant	N11 For Lib	N11 Rev Lib
R12 Double Mutant	R12 For Lib	R12 Rev Lib
P13 Double Mutant	P13 For Lib	P13 Rev Lib
M14 Double Mutant	M14 For Lib	M14 Rev Lib
H15 Double Mutant	H15 For Lib	H15 Rev Lib
I16 Double Mutant	I16 For Lib	I16 Rev Lib
H17 Double Mutant	H17 For Lib	H17 Rev Lib
D18 Double Mutant	D18 For Lib	D18 Rev Lib
W19 Double Mutant	W19 For Lib	W19 Rev Lib
Q20 Double Mutant	Q20 For Lib	Q20 Rev Lib
I21 Double Mutant	I21 For Lib	I21 Rev Lib

M22 Double Mutant	M22 For Lib	M22 Rev Lib
D23 Double Mutant	D23 For Lib	D23 Rev Lib

Table S10. HPLC collection windows.

Ubonodin Variant	HPLC Round 1 Collection Window	HPLC Round 2 Collection Window	Solvent for Dissolving Pure Peptide
A7P	15.32 min-15.55 min	10.65 min – 11.2 min	water
R12F	17.15 min-17.7 min	N/A	50/50 water/acetonitrile
M14N	13.95 min – 14.2 min	7.5 min - 7.8 min	water
I16D	13.8 min – 14.2 min	7. 45 min – 7.9 min	water
I16E	13.8 min - 14.15 min	7.55 min - 8 min	water
H17G	15.8 min – 16.1 min	N/A	50/50 water/acetonitrile
A7G N11M	15.8 min – 16.05 min	N/A	50/50 water/acetonitrile
S5H I16P	13.6 min – 14 min	21.5 min – 21.8 min	water
A7P I16A	14.15 min-14.8 min	N/A	water
R12F W19G	15.9 min-16.5 min	13.15 min -14.8 min	50/50 water/acetonitrile
S5T I16D	13.9 min – 14.3 min	7.6 min – 8 min	water
I16E D23A	13.8 min – 14.3 min	7.2 min – 7.8 min	water
R12V H17G	17.4 min -17.8 min	N/A	50/50 water/acetonitrile

References

1. Broom, B. M.; Ryan, M. C.; Brown, R. E.; Ikeda, F.; Stucky, M.; Kane, D. W.; Melott, J.; Wakefield, C.; Casasent, T. D.; Akbani, R.; Weinstein, J. N., A Galaxy Implementation of Next-Generation Clustered Heatmaps for Interactive Exploration of Molecular Profiling Data. *Cancer Res.* **2017,** *77* (21), e23-e26.
2. Li, F.; Yuan, L.; Lu, H.; Li, G.; Chen, Y.; Engqvist, M. K. M.; Kerkhoven, E. J.; Nielsen, J., Deep learning-based kcat prediction enables improved enzyme-constrained model reconstruction. *Nat. Catal.* **2022,** *5* (8), 662-672.
3. Braffman, N. R.; Piscotta, F. J.; Hauver, J.; Campbell, E. A.; Link, A. J.; Darst, S. A., Structural mechanism of transcription inhibition by lasso peptides microcin J25 and capistruin. *Proc. Natl. Acad. Sci. U. S. A.* **2019,** *116* (4), 1273-1278.
4. Acevedo-Rocha, C. G.; Reetz, M. T.; Nov, Y., Economical analysis of saturation mutagenesis experiments. *Sci. Rep.* **2015,** *5*, 10654.
5. Cheung-Lee, W. L.; Parry, M. E.; Jaramillo Cartagena, A.; Darst, S. A.; Link, A. J., Discovery and structure of the antimicrobial lasso peptide citrocin. *J Biol. Chem.* **2019,** *294* (17), 6822-6830.
6. Metelev, M.; Arseniev, A.; Bushin, L. B.; Kuznedelov, K.; Artamonova, T. O.; Kondratenko, R.; Khodorkovskii, M.; Seyedsayamdost, M. R.; Severinov, K., Acinetodin and Klebsidin, RNA Polymerase Targeting Lasso Peptides Produced by Human Isolates of Acinetobacter gyllenbergii and Klebsiella pneumoniae. *ACS Chem. Biol.* **2017,** *12* (3), 814-824.
7. Carson, D. V.; Patino, M.; Elashal, H. E.; Cartagena, A. J.; Zhang, Y.; Whitley, M. E.; So, L.; Kayser-Browne, A. K.; Earl, A. M.; Bhattacharyya, R. P.; Link, A. J., Cloacaenodin, an Antimicrobial Lasso Peptide with Activity against Enterobacter. *ACS Infect. Dis.* **2023,** *9* (1), 111-121.
8. Becka, S. A.; Zeiser, E. T.; LiPuma, J. J.; Papp-Wallace, K. M., Activity of Imipenem-Relebactam against Multidrug- and Extensively Drug-Resistant Burkholderia cepacia Complex and Burkholderia gladioli. *Antimicrob. Agents Chemother.* **2021,** *65* (11), e0133221.
9. Papp-Wallace, K. M.; Becka, S. A.; Zeiser, E. T.; Ohuchi, N.; Mojica, M. F.; Gatta, J. A.; Falleni, M.; Tosi, D.; Borghi, E.; Winkler, M. L.; Wilson, B. M.; LiPuma, J. J.; Nukaga, M.; Bonomo, R. A., Overcoming an Extremely Drug Resistant (XDR) Pathogen: Avibactam Restores Susceptibility to Ceftazidime for Burkholderia cepacia Complex Isolates from Cystic Fibrosis Patients. *ACS Infect. Dis.* **2017,** *3* (7), 502-511.
10. Zeiser, E. T.; Becka, S. A.; Wilson, B. M.; Barnes, M. D.; LiPuma, J. J.; Papp-Wallace, K. M., "Switching Partners": Piperacillin-Avibactam Is a Highly Potent Combination against Multidrug-Resistant Burkholderia cepacia Complex and Burkholderia gladioli Cystic Fibrosis Isolates. *J. Clin. Microbiol.* **2019,** *57* (8).
